# Long-term decrease in Asian monsoon rainfall and abrupt climate change events over the past 6,700 years

**DOI:** 10.1073/pnas.2102007118

**Published:** 2021-07-19

**Authors:** Bao Yang, Chun Qin, Achim Bräuning, Timothy J. Osborn, Valerie Trouet, Fredrik Charpentier Ljungqvist, Jan Esper, Lea Schneider, Jussi Grießinger, Ulf Büntgen, Sergio Rossi, Guanghui Dong, Mi Yan, Liang Ning, Jianglin Wang, Xiaofeng Wang, Suming Wang, Jürg Luterbacher, Edward R. Cook, Nils Chr. Stenseth

**Affiliations:** ^a^Key Laboratory of Desert and Desertification, Northwest Institute of Eco-Environment and Resources, Chinese Academy of Sciences, Lanzhou 730000, China;; ^b^Chinese Academy of Sciences Center for Excellence in Tibetan Plateau Earth Sciences, Chinese Academy of Sciences, Beijing 100101, China;; ^c^Institute of Geography, Friedrich-Alexander-University Erlangen-Nürnberg, 91058 Erlangen, Germany;; ^d^Climatic Research Unit, School of Environmental Sciences, University of East Anglia, Norwich NR4 7TJ, United Kingdom;; ^e^Laboratory of Tree-Ring Research, University of Arizona, Tucson, AZ 85721;; ^f^Department of History, Stockholm University, 106 91 Stockholm, Sweden;; ^g^Bolin Centre for Climate Research, Stockholm University, 106 91 Stockholm, Sweden;; ^h^Swedish Collegium for Advanced Study, 752 38 Uppsala, Sweden;; ^i^Department of Geography, Johannes Gutenberg University, 55099 Mainz, Germany;; ^j^Global Change Research Institute of the Czech Academy of Sciences (CzechGlobe), 603 00 Brno, Czech Republic;; ^k^Department of Geography, Justus-Liebig-University, D-35390 Giessen, Germany;; ^l^Department of Geography, University of Cambridge, CB2 3EN Cambridge, United Kingdom;; ^m^Dendrosciences Group, Swiss Federal Research Institute for Forest, Snow and Landscape Research, CH-8903 Birmensdorf, Switzerland;; ^n^Czech Globe Global Change Research Institute, Czech Academy of Sciences, 60300 Brno, Czech Republic;; ^o^Department of Geography, Faculty of Science, Masaryk University, 61137 Brno, Czech Republic;; ^p^Département des Sciences Fondamentales, Université du Québec à Chicoutimi, Chicoutimi, QC G7H 2B1, Canada;; ^q^Key Laboratory of Vegetation Restoration and Management of Degraded Ecosystems, Guangdong Provincial Key Laboratory of Applied Botany, South China Botanical Garden, Chinese Academy of Sciences, Guangzhou 510650, China;; ^r^College of Earth and Environmental Sciences, Lanzhou University, Lanzhou 730000, China;; ^s^Key Laboratory of Virtual Geographic Environment of Ministry of Education, School of Geography Science, Nanjing Normal University, Nanjing 210023, China;; ^t^State Key Laboratory of Lake Science and Environment, Nanjing Institute of Geography and Limnology, Chinese Academy of Sciences, Nanjing 210008, China;; ^u^Science and Innovation Department, World Meteorological Organization, CH-1211 Geneva, Switzerland;; ^v^Tree Ring Laboratory, Lamont Doherty Earth Observatory of Columbia University, Palisades, NY 10964;; ^w^Centre for Ecological and Evolutionary Synthesis, Department of Biosciences, University of Oslo, N-0316 Oslo, Norway

**Keywords:** tree rings, stable isotopes, climate variability, megadrought, Asian summer monsoon

## Abstract

Asian summer monsoon (ASM) variability and its long-term ecological and societal impacts extending back to Neolithic times are poorly understood due to a lack of high-resolution climate proxy data. Here, we present a precisely dated and well-calibrated tree-ring stable isotope chronology from the Tibetan Plateau with 1- to 5-y resolution that reflects high- to low-frequency ASM variability from 4680 BCE to 2011 CE. Superimposed on a persistent drying trend since the mid-Holocene, a rapid decrease in moisture availability between ∼2000 and ∼1500 BCE caused a dry hydroclimatic regime from ∼1675 to ∼1185 BCE, with mean precipitation estimated at 42 ± 4% and 5 ± 2% lower than during the mid-Holocene and the instrumental period, respectively. This second-millennium–BCE megadrought marks the mid-to late Holocene transition, during which regional forests declined and enhanced aeolian activity affected northern Chinese ecosystems. We argue that this abrupt aridification starting ∼2000 BCE contributed to the shift of Neolithic cultures in northern China and likely triggered human migration and societal transformation.

## Significance

The variability of the Asian summer monsoon (ASM) is important for the functioning of ecological and societal systems at regional to continental scales, but the long-term evolution and interannual variability of this system is not well understood. Here, we present a stable isotope–based reconstruction of ASM variability covering 4680 BCE to 2011 CE. Superimposed on a gradual drying trend, a rapid drop in mean annual precipitation (>40%) toward persistently drier conditions occurred in ∼1675 BCE. This megadrought caused regional forest deterioration and enhanced aeolian activity affecting Chinese ecosystems. We argue that this abrupt aridification starting ∼2000 BCE triggered waves of human migration and societal transformation in northern China, which contributed to the alteration of spatial pattern of ancient civilizations.

Climatic change and variability can have large and long-lasting consequences for ecosystems and human societies (1–7). Despite a complex interplay of environmental and nonenvironmental factors, favorable (e.g., warm and wet) climatic conditions have been globally linked to the rise of civilizations, whereas unfavorable conditions have been associated with social instability, human migration, and the more-frequent transformation of civilizations (8–19). The paucity of high-resolution climate proxy archives that extend prior to the CE, however, prevents a detailed analysis of the linkages between climate variability and potential societal responses for this early period. This is particularly the case for the vast region influenced by the Asian summer monsoon (ASM), for which a good coverage of archaeological data exists that potentially can be used to link climate variability with societal change far back in time.

**Fig. 1.**
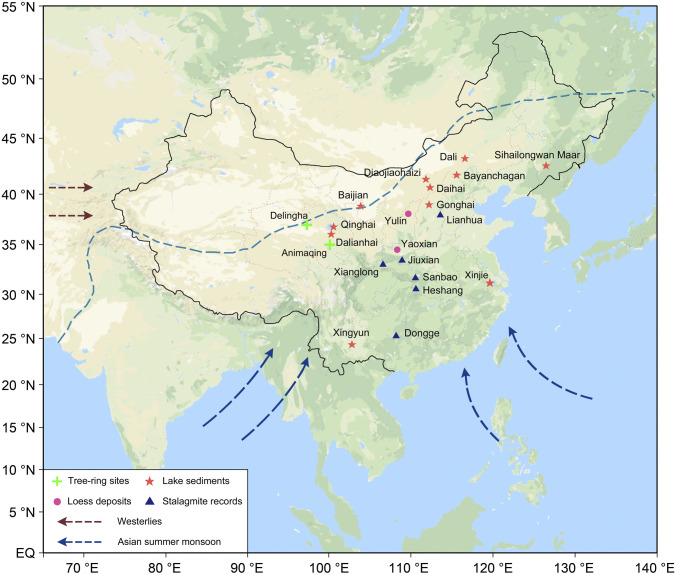
Locations of Holocene paleoclimate records included in this study. The arrows depict the ASM and the Westerlies. The blue dashed line indicates the approximate present-day northern extent of the ASM region based on the observed mean 2 mm/d summer isohyet after ref. 52. The blue triangles represent stalagmite records, the purple dots indicate loess-paleosol profiles, the red asterisks indicate lake sediment records, and the green crosses indicate tree-ring chronologies (including DLH). See *SI Appendix*, Table S6 for details about each paleoclimate record.

**Fig. 2.**
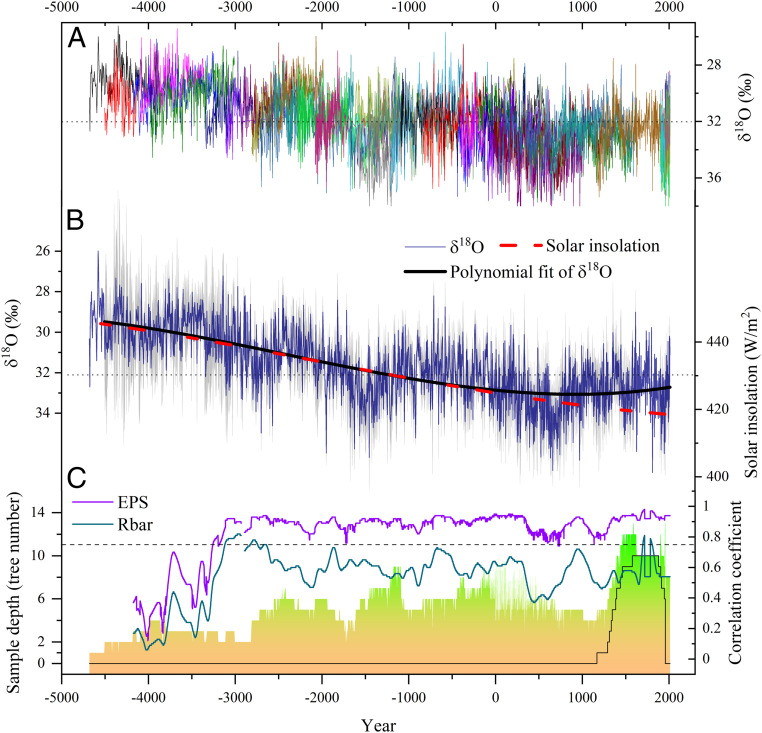
The DLH tree-ring δ^18^O chronology. (*A*) Visualization of all 44 δ^18^O measurement series. (*B*) DLH δ^18^O chronology (navy blue line), third-order polynomial fitting of this chronology (thick black line), and July solar insolation between 30°N and 60°N (red line). The gray shading indicates the 95% CI of the composite δ^18^O chronology. For better comparison, the *y*-axis of the δ^18^O chronology was reversed. (*C*) Sample depth (with the black line indicating the number of trees in the pooled series) of the DLH δ^18^O chronology and Rbar (gray line) and EPS (purple line) of the δ^18^O dataset, calculated over a 250-y window in steps of 1 y. The Rbar time series was smoothed with a 100-y Gaussian-weighted filter. The annual values with EPS ≥ 0.85 accounts for 80.2% during 3250 BCE to 2011 CE, whereas 91.2% of values have EPS ≥ 0.25 and 37.7% are ≥ 0.50 before 3250 BCE.

Here, we present an exactly calendar-year dated (by dendrochronological cross-dating) tree-ring–based stable oxygen isotope chronology (the Delingha [DLH] δ^18^O chronology, Figs. 1 and 2) covering ∼6,700 y from 4680 BCE to 2011 CE, which represents the longest existing precisely dated isotope chronology in Asia. In this chronology, we combined stable isotope series from 53 living and relict trees from the DLH region on the northeastern Tibetan Plateau (TP) (Fig. 1), based on a total of 9,526 isotope measurements (*SI Appendix*, Materials and Methods). The agreement in point-to-point variability between individual tree-ring samples (Fig. 2 *A* and *C*) demonstrates the reliability of this composite mean isotope chronology.

**Fig. 3.**
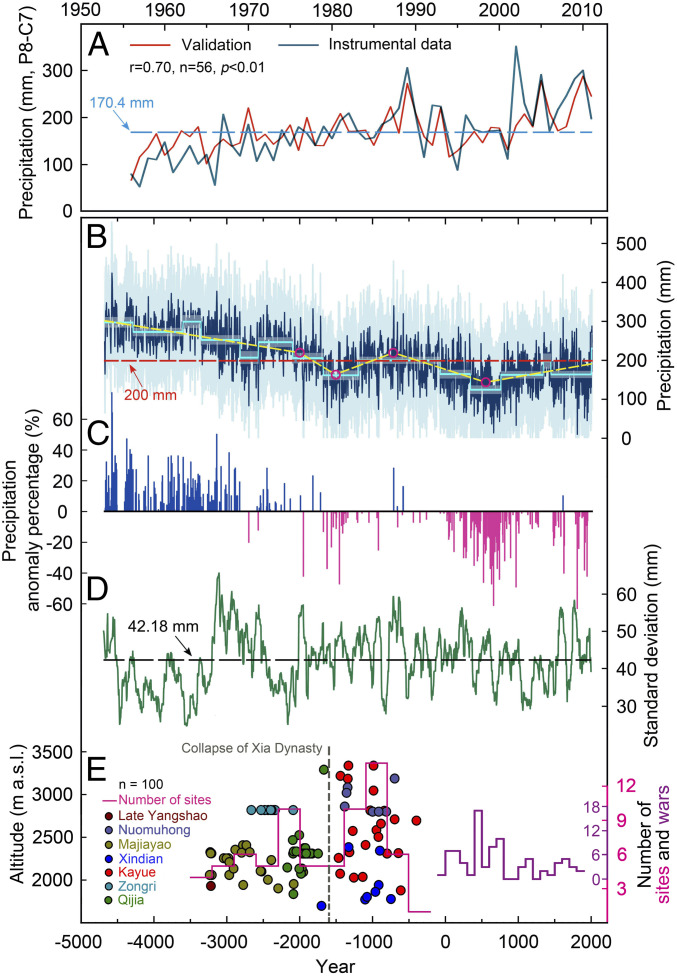
Annual (prior August to current July) tree-ring δ^18^O precipitation reconstruction ranging from 4680 BCE to 2011 CE. (*A*) Comparison between reconstructed (red) and instrumental (blue) precipitation (1956 to 2011 CE). The horizontal dashed line indicates the annual mean precipitation (170.4 mm) over the instrumental period (1956 to 2011 CE). (*B*) Reconstructed precipitation (blue) and 95% CIs (light blue shading). The sky-blue step lines represent regime shifts, and the associated shading indicates 95% CIs for each subperiod (*SI Appendix*, Materials and Methods). Significant changes in temporal trends (yellow line, with magenta circles indicating trend change point years with *P* < 0.05: 544 CE, 709 BCE, 1501 BCE, and 2000 BCE; *SI Appendix*, Materials and Methods). The red horizontal line is the reconstructed mean precipitation of the entire period (4680 BCE to 2011 CE). (*C*) Extreme dry and wet annual events 4680 BCE to 2011 CE. The events were identified in the precipitation reconstruction as those years in which the precipitation exceeded the 10th and 90th percentiles of the whole period and expressed as percent anomalies from the instrumental period mean. (*D*) The 100-y running SD of the reconstructed mean annual precipitation. (*E*) Prehistoric cultural responses to rapid climatic change on the northeastern TP and in northern China (47, 53). The dots of different colors indicate calibrated accelerator mass spectrometry dates of charred grains and bones unearthed from Neolithic and Bronze sites on the northeastern TP, while the pink step line represents temporal variations of number of dated sites every 300 y. The purple step line denotes variations of war frequency over time in east Qinghai Province during the past two millennia (32, 33).

The DLH region is situated at the present-day northwestern fringe of the ASM region (Fig. 1), and our tree-ring record sensitively reflects temporal changes in ASM intensity (*SI Appendix*, Figs. S16 and S17). Due to the current arid conditions (mean annual precipitation of 170.4 mm, about 85% of which falls in summer [May to September]), tree growth in this region is strongly controlled by precipitation (20). Via soil moisture, precipitation variability controls δ^18^O ratios in tree-ring cellulose, which is confirmed by the fact that 49% of the variance in annual instrumental precipitation data (prior August to current July; 1956 to 2011) is accounted for by the DLH δ^18^O chronology. This strong relationship, confirmed by leave-one-out cross-validation (Fig. 3*A*), allows us to reconstruct regional hydroclimate variability with an unprecedented detail with a 5-y minimum resolution over the past ∼6,700 y (Fig. 3 *B*–*D*).

**Fig. 4.**
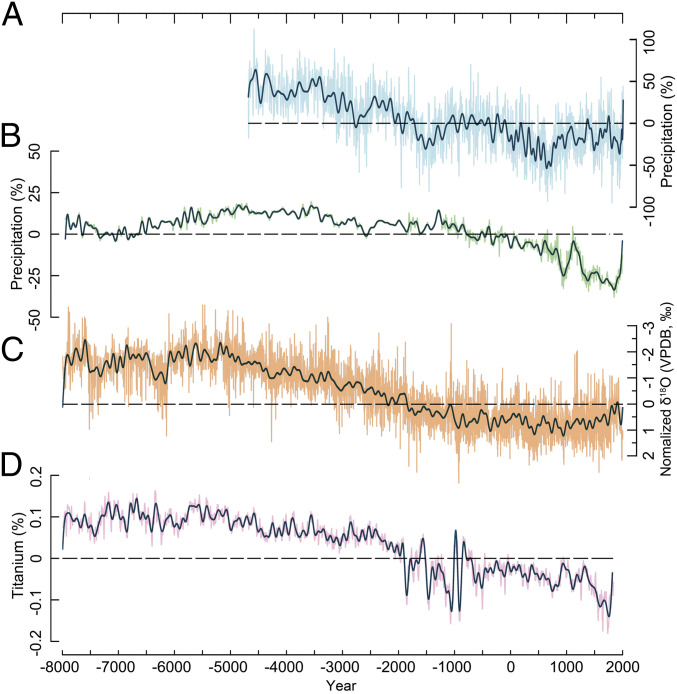
Comparison of the DLH tree-ring δ^18^O precipitation reconstruction with other paleoclimatic records spanning the Holocene. (*A*) Anomaly percentage of the DLH precipitation reconstruction calculated over the period 4680 BCE to 1950 CE (this study). (*B*) Pollen-based annual precipitation anomaly percentage in Gonghai Lake calculated over the common period 4680 BCE to 1950 CE (24). (*C*) Normalized stalagmite composite δ^18^O record from eastern China. The *y*-axis of the composite δ^18^O record was reversed for better comparison. Each stalagmite δ^18^O record was first normalized over the common period 4700 BCE to 1300 CE using the equation (*a* − *b*_*m*_) / *b*_*s*_, where *a* is the original value, and *b*_*m*_ and *b*_*s*_ are the mean and SD of the common period, respectively. See *SI Appendix*, Table S6 (site no.: 1 to 6) for details about each stalagmite record employed in the calculation. (*D*) Variation in location of the ITCZ reflected by Cariaco Basin Ti concentrations (26). All horizontal lines represent the long-term average calculated over the common period 4680 BCE to 1950 CE. The long-term precipitation average values are 200 and 511 mm, respectively for panels (*A* and *B*). For panels (*A*–*D*), all series were first interpolated annually by using a piecewise linear interpolation method, and then each series (thin line) was smoothed by a 100-point low-pass filter (heavy line) to highlight the centennial scale variability.

Our precipitation reconstruction shows a pronounced multimillennial drying trend (Figs. 3*B* and 4*A*). This trend is in agreement with proxy evidence of lower temporal resolution from stalagmite δ^18^O records from eastern China (21–23), pollen-based precipitation reconstructions from eastern China (24), and other moisture-sensitive proxy archives (Figs. 1 and 4 *B* and *C*, and *SI Appendix*, Figs. S12–S15). However, our DLH reconstruction quantifies long- and short-term climatic events at a much higher temporal resolution and with precise dating accuracy, offering a unique benchmark record to synchronize Chinese archaeological evidence and anchor a range of contemporary paleoenvironmental data. It also benefits from a robust calibration between the climate proxy and instrumental climatic data, and an in-depth comparison with model simulations.

A long-term aridification trend since the mid-Holocene is evident, which closely matches a corresponding negative trend in summer solar insolation from 30 to 60°N (Figs. 2*B* and3*B*). Thus, we hypothesize that summer insolation has been a primary driver of long-term aridification at the northern limits of the ASM zone of China since the mid-Holocene. Decreasing summer insolation may have considerably reduced the thermal contrast between the Asian continent and the surrounding oceans, thereby leading to a displacement of the Intertropical Convergence Zone (ITCZ) and a weakening of the ASM circulation resulting in reduced precipitation in the ASM marginal areas.

The long-term aridification that characterizes our DHL reconstruction and other proxy evidence (*SI Appendix*, Fig. S15), accompanied by the cooling trend through the middle to late Holocene, is confirmed by the CCSM3 climate model (*SI Appendix*, Materials and Methods) that simulates decreasing temperature and precipitation trends in northern China (25). Our precipitation reconstruction is positively correlated with centennial-scale China-wide temperature variability over the most recent two millennia (*SI Appendix*, Fig. S18), suggesting that future large-scale warming might be associated with even greater moisture supply in this region. Model simulations also suggest that the long-term moisture variations in the marginal monsoon region are closely linked to shifts in the mean position of the ITCZ, as also indicated by titanium concentration trends from the Cariaco Basin in the Caribbean Sea (26) (Fig. 4*D*).

In addition to temporal ASM variability, the mean DLH δ^18^O value can also reflect changes in spatial ASM extent. We compared the mean δ^18^O value of our DLH chronology with another Qilian juniper isotope chronology from the Animaqing Mountains located 300 km to the southeast of our study site at a similar elevation. For the recent period (1930 to 2011 CE), δ^18^O in Animaqing amounts to 30.78 ± 1.33‰ (27), which is significantly lower than at DLH (32.84 ± 1.07‰). However, the mean value in the earliest part of our DLH δ^18^O chronology (4680 to 3000 BCE; 29.80 ± 1.12‰) is closer to the present-day Animaqing values, indicating that humid present-day climate conditions in the Animaqing Mountains may be used as a modern analog for mid-Holocene climate in the DLH region. Given this, we infer that during the mid-Holocene, the ASM limit extended at least 300 km further northwest compared to its present-day limit.

An assumed northward shift of the ASM boundary during the mid-Holocene is supported by additional regional paleoclimatic evidence of lower temporal resolution. A 300- to 400-km northwestward migration of the ASM rain belt during the early and mid-Holocene has been suggested from a lake size record from northeastern China (28) and from plant biomass data in loess sections across the Loess Plateau (29). A climate reconstruction combining vegetation type and sedimentary facies in aeolian deposits (30) further suggests that deserts in northern China retreated by ∼200 km to the northwest during the mid-Holocene (4800 ± 300 BCE).

Our high-resolution precipitation reconstruction provides absolute estimates for precipitation differences between the mid-Holocene and present-day conditions. We estimate mean annual precipitation during the mid-Holocene (here, 4680 to 3000 BCE) as 279 ± 10 mm, which exceeds the average levels of the entire reconstruction period (4680 BCE to 2011 CE; 200 ± 9 mm) and of the instrumental period (1956 to 2011 CE; 170.4 mm) by 40 (∼38 to 41% at 95% confidence) and 63% (∼57 to 69% at 95% confidence), respectively (Figs. 3*B* and 4*A*).

Our precipitation reconstruction also reveals centennial-scale variability that differs substantially from a ∼20-y–resolution pollen-based annual precipitation record (24) (Fig. 4 *A* and *B*). In comparison with this pollen-based reconstruction, which shows precipitation variations in the range of ±25% of the long-term average, the DLH δ^18^O reconstruction displays a much larger centennial-scale variability, ranging from −50 to 50%.

Using a sequential Student’s *t* test approach, we identified several major, clearly dateable centennial-scale regime shifts (Fig. 3*B* and *SI Appendix*, Fig. S10 and Table S7) in our DLH record (31) (*SI Appendix*, Materials and Methods). We detected the strongest shifts toward dry conditions around 3350, 2815, 2095, 1675, and 70 BCE and 346 CE (*SI Appendix*, Table S7). Regime shifts toward wetter conditions were typically less dramatic, and occurred in 2565, 1185 BCE, and 760 CE (*SI Appendix*, Table S5). The precise dating of these regime shifts allows us to determine the duration and magnitude of past dry epochs.

The most severe and long-lasting dry period prior to the CE occurred c. 1675 to 1185 BCE (Fig. 3*B* and *SI Appendix*, Table S7), representing a remarkable megadrought (mainly represented on a millennial scale with three obvious centennial droughts superimposed, *SI Appendix*, Fig. S11) with an estimated mean annual precipitation of 42 ± 4 and 5 ± 2% less than the average over the mid-Holocene (4680 to 3000 BCE) and the instrumental period (1956 to 2011 CE), respectively. Trend-point analysis (*SI Appendix*, Fig. S10) confirms that this 1675 to 1185 BCE megadrought marks a low in the long-term general drying trend in the DLH reconstruction, which intensified between ∼2000 and ∼1500 BCE (Fig. 3*B*). This period of rapidly decreasing moisture availability starting ∼2000 BCE and culminating ∼1500 BCE thus arguably marks the transition from the mid- to the late Holocene Asian moisture regime.

Another period of long-lasting extremely dry conditions occurred c. 346 to 763 CE (Fig. 3*B* and *SI Appendix*, Table S7). This extremely dry period, when war frequency reached a maximum in east Qinghai Province due to conflicts between different local regimes and decreased rapidly afterward (32, 33) (Fig. 3*E*), was also recorded in other hydroclimatic proxies in China (20) and partly overlaps with the “Late Antique Little Ice Age” (LALIA) (2). The correspondence of social unrest and drought indicates a likely impact of climate deterioration on society at that time. At a hemispheric scale, Zhang et al. (34) argued that climate change may have imposed a spatially wider-ranging effect on human civilization.

The LALIA megadrought represents the culmination of the millennial-scale drying trend in the DLH reconstruction, which reversed around ∼544 CE (indicated by trend-point analysis; *P* < 0.05; *SI Appendix*, Fig. S10 and Fig. 3*B*). As a result of this hydroclimatic trend reversal, precipitation and insolation trends started to diverge by the middle of the first millennium CE, when solar insolation continued to decrease, whereas precipitation did not (Figs. 2*B* and 3*B*).

Our mid-Holocene–length hydroclimate reconstruction thus records multiple distinct climate regime shifts. However, it does not support a significant transition in the hydroclimate of our study region around ∼2200 BCE during the so-called “4.2-ka event” (35), nor the notion that this rapid climate deterioration and associated global-scale megadroughts should be regarded as a generalized climatic transition from the mid- to late Holocene (36).

At high temporal resolution, our DLH reconstruction shows that moisture conditions alternated between extremely wet and dry periods at interannual, decadal, and multidecadal timescales (Fig. 3*B* and *SI Appendix*, Table S8). For example, mean annual precipitation extremes of opposite signs can occur within a few decades (e.g., 309 mm in 1990 BCE compared with 47 mm in 1950 BCE and 313 mm in 1715 BCE compared with 95 mm in 1675 BCE). In the most recent 50 y (1956 to 2011), precipitation has increased in our study region and had previously been found to be the wettest period of the past 3,500 y (20). However, our DHL precipitation reconstruction indicates that this wet recent period is not unprecedented in historical times (Fig. 3*B*). The discrepancy between the two studies can likely be attributed to the strength of the precipitation signal in the two tree-ring parameters (tree-ring width in ref. 20 versus δ^18^O in this study), the extension of the DLH δ^18^O chronology into the wetter mid-Holocene, and concerns about whether the detrended tree-ring width record (20) is able to capture climate variability on millennial timescales (*SI Appendix*, Fig. S12).

Wet extremes occurred with the highest intensity and frequency prior to 2800 BCE (Fig. 3*C* and *SI Appendix*, Tables S3 and S8). In line with the long-term aridification trend, the frequency and magnitude of wet extremes in our record decreased over the following two millennia. In contrast, the frequency of dry extremes increased and peaked around 660 CE, with potentially harmful impacts on contemporary human societies.

Precipitation variability has changed considerably over time, as shown by a 100-y running SD plot (Fig. 3*D*). Over the entire record, the mean SD is 42 mm, but extended periods of low SD occurred from 4680 to 3200 BCE, 2500 to 2000 BCE, and 1000 to 1500 CE. The first of these is particularly notable because of the sudden transition toward a period with particularly high variability around 3200 BCE.

The humid climate during the mid-Holocene and the subsequent aridification had major impacts on the ecological environment in China. Pollen records from northern China testify to a broad-scale transition from forest to steppe vegetation in the climate-sensitive ASM margin around ∼1600 BCE (37) (*SI Appendix*, Fig. S19). In the more humid eastern TP, a phase of major deterioration of *Picea* forests occurred after 1600 BCE. Woody debris in Qinghai Lake sediments verify that spruce (*Picea crassifolia* Kom.) forests had already developed in the region 7700 to 2200 BCE and subsequently disappeared (38). Combining these results with our ASM reconstruction, we propose that wetter conditions during the mid-Holocene played a major role in establishing a denser regional forest cover. The subsequent abrupt aridification (reaching a very dry regime by ∼1675 BCE) initiated a broad-scale forest decline in northern China, finally resulting in the disappearance of spruce forests in the Qinghai Lake basin. The mid- to late Holocene aridification trend is also reflected by enhanced aeolian activity (39).

Our DLH precipitation reconstruction supports assessments of the societal responses to rapid climatic change in China. The wet and climatically rather stable mid-Holocene (Fig. 3 *B* and *D*) likely contributed to facilitate the expansion of the Yangshao culture across China (Fig. 3*E*). The prosperity of the Majiayao (3300 to 2000 BCE) and Qijia cultures (2300 to 1600 BCE) in the Gansu-Qinghai region (40–43) may also be associated with contemporary favorable regional climate conditions. In the northern and southern Loess Plateau, two large-scale Neolithic urban centers, Shimao (2300 to 1800 BCE) and Taosi (2300 to 1900 BCE), flourished (44, 45). Both centers were abandoned after 1800 BCE, perhaps partly as a result of the rapid regime shift from a wet to a dry climate in the second-millennium BCE (considering the radiocarbon dating uncertainty of the archaeological material).

This second-millennium–BCE megadrought may also have had a major impact on human civilizations in the semiarid and arid regions of northern China, where water availability is a major constraint for human subsistence. A sudden drop in the number of archaeological sites on the northeastern TP occurred between 2000 and 1400 BCE, as shown by calibrated accelerator mass spectrometry radiocarbon dates of charred grains and bones (Fig. 3*E*). The Qijia culture began to disintegrate around 1600 BCE and evolved into multiple cultures (e.g., Kayue, Xindian, and Nuomuhong) (Fig. 3*E*). Such dry and cold climate along with increased climate variability (Fig. 3*D*), coupled with innovations in agriculture, could have contributed to the process and led to a change in a subsistence strategy from millet farming to combined barley and wheat farming in the Gansu-Qinghai region (46). Substituting millet production with barley that is better adapted to the cooler and drier conditions likely limited the risk of crop failure and enabled humans to cultivate at TP altitudes above 3,000 m above sea level (43, 46, 47). After ∼1500 BCE, barley spread southwards into the southeastern TP and replaced millet that could not adapt to cooler and drier conditions of the late Holocene (48). Meanwhile, in the western Loess Plateau, human subsistence went through a major transition from long-established rain-fed agriculture to mobile pastoralism after ∼1600 BCE (42, 49), which is consistent with the c. 1675 to 1190 BCE megadrought recorded in our precipitation reconstruction.

The effects of the second-millennium–BCE megadrought become apparent in a comprehensive review of archaeological evidence across China, including 51,074 sites covering most parts of China and spanning the early Neolithic to early Iron Age (c. 8000 to 500 BCE) (50, 51). Herein, a steady increase in the number of archaeological sites can be detected from 5800 to 1750 BCE (50), implying continuous cultural development in large areas of China. The absence of evidence for irrigation-based farming indicates that rain-fed agriculture was sufficient to sustain Neolithic and early Chalcolithic communities (52). The abrupt aridification around 1675 BCE corresponded to a sudden reduction in the number of archaeological sites, as well as a contraction in the areal distribution of sites across all of China (*SI Appendix*, Fig. S20). The number of archaeological sites around the middle and lower reaches of the Yellow River decreased substantially, marking the almost-complete abandonment of the Guanzhong Basin (51), while the highest number of sites during this period can be found in northeastern China (50, 51). Therefore, it seems that the aridification around 2000 to 1500 BCE could be, at least partly, responsible for a large human migration phase in northern China. At the same time (2000 to 1600 BCE), the earliest documented Chinese kingdoms associated with the Xia dynasty emerged, which were later replaced by the Shang dynasty (∼1600 to 1000 BCE) (53). In view of all the evidence stated above, we propose that the second-millennium–BCE megadrought might have accelerated the disintegration of these historical civilizations.

In conclusion, we present a precisely dated benchmark timeseries representing multiscale variability in ASM intensity and extent over the past 6,700 y. We show that solar insolation is responsible for driving most of the multimillennial variation in ASM intensity. We identified two severe and long-lasting dry periods, 1675 to 1185 BCE and 346 to 763CE, that both correspond to periods of regional societal turbulence. We propose that rapidly decreasing moisture availability starting ∼2000 BCE marks the transition from mid- to late Holocene and resulted in unfavorable environmental conditions, ultimately exerting severe pressures on natural forest vegetation, crop production, and societal development in northern China. These cultures collapsed one by one, initiated around ∼2000 BCE by the aridification of the local climate. In this context, some of the extreme drought events recorded by our reconstruction might have accelerated the disintegration of ancient civilizations. The complexity of their social structure, associated with differing adaptation abilities and strategies to resist adverse climatic stress, can explain regional differences in timing of their disintegration.

## Materials and Methods

### Sample Collection and δ^18^O Chronology Development.

Tree samples were collected from two open canopy sites in the DLH region on the northeastern TP. The two sites, MNT (37.45°N to 37.46°N, 97.67°E to 97.69°E) and QK (37.46°N to 37.48°N, 97.77°E to 97.78°E), represent two generally homogeneous growth environments in close proximity, located less than 30 km apart. These juniper trees can reach ages over 3,000 y old, and living trees over 2,000 y old are not unusual (20, 54, 55). We selected a total of 53 tree samples (39 dead trees and 14 living trees) that met the criteria of normal growth, clear ring boundaries, and few missing rings, for the subsequent δ^18^O measurements. The most recent ring from a dead tree sample dated to 1943 CE. We did not use any archaeological wood samples in this study. In summary, 9,526 individual ring samples were analyzed to obtain the full δ^18^O series. We conducted experiments and sensitivity tests to investigate four potential nonclimatic influences on the δ^18^O measurements: sampling altitude, age-related trends, juvenile effects, and outlier values (see *SI Appendix*, Materials and Methods for details). Altitude and juvenile effects on tree-ring δ^18^O were examined and found to be negligible, and local age-related influences on tree-ring cellulose δ^18^O were not observed in the study area. The latest studies on European oak stable oxygen isotope measurements confirmed the absence of age trends in time series of this tree-ring parameter (56–59). We thus developed a merged δ^18^O chronology spanning from 4680 BCE to present based on the arithmetic mean of all the δ^18^O series in the same calendar year. The expressed population signal (EPS) was calculated for 250-y intervals shifted along the chronology in steps of 1 y to estimate temporal changes in signal strength related to declining sample replication (see *SI Appendix*, Materials and Methods for details). As pointed out by Wigley et al. (60), EPS has no strict significance threshold and is best used simply as a guide for interpreting the changing level of uncertainty in a mean series as its statistical signal strength changes over time.

Level offsets (i.e., differences in the means) in the tree-ring δ^18^O time series of different trees could result in a bias when combining individual δ^18^O series into a composite chronology (61–64). Sensitivity tests, in which we compared results with inclusion and exclusion of extreme mean tree-ring δ^18^O series and compared the mean and median of the δ^18^O values in each year, show that the offsets between the means of individual tree-ring δ^18^O time series have a small influence on the interannual and even decadal scales. This influence, however, is negligible on multidecadal, centennial, and multimillennial scales (*SI Appendix*, Fig. S7). We used, therefore, the entire mean chronology for analysis, even though we note that the EPS is not high in the early part (4680 to 3250 BCE) of the chronology when the sample replication is low (Fig. 2*C*). Nevertheless, it is clear (Fig. 2*A*) that the level of the individual tree-ring δ^18^O series is unusually low during 4680 to 3250 BCE, characterized by persistently wet conditions. In particular, almost no values (except for one) are higher than the long-term mean of the mean δ^18^O chronology. This consistency demonstrates that the mean of the individual δ^18^O series represents a real climate signal.

### Climate Calibration.

Since ordinary regression analysis showed that regression residuals were significantly autocorrelated (lag-1 autocorrelation = 0.38, *P* < 0.01) over time, thus violating the assumption that the errors are independent of each other, a first-order autoregressive model (AUTOREG) was applied to reconstruct the annual (prior August to current July) precipitation of the past 6,700 y (*SI Appendix*, Materials and Methods). The annual precipitation reconstruction explains 49% (*n* = 56, *P* < 0.01) of the variance in the DLH instrumental precipitation record. We initially used a “leave-one-out” cross-validation procedure to evaluate the statistical fidelity of our reconstruction model by using the AUTOREG model. The test statistic reduction of error (RE) has a positive value of 0.44, verifying the statistical validity of our reconstruction model. In addition, we calculated a standard split-period calibration-verification test to evaluate the statistical skill of our reconstruction model. The resulting statistics are shown in *SI Appendix*, Table S5. The RE and the coefficient of efficiency values are positive and the results of the sign test, which describes how well the predicted value tracks the direction of the observed data, exceed the 95% confidence level. These test results confirm the skill of our reconstruction model. The uncertainty ranges for the average precipitation of some subperiods of the entire reconstruction series were calculated with a modification factor multiplying the ±1 RMSE (root mean square error) since uncertainty ranges are timescale dependent (65). The modification factor is defined as Gamma / sqrt(*n*), where Gamma = (1 + r) / (1 − r), with r being the lag-1 autocorrelation coefficient of the residual time series and *n* the number of years used for the average.

### Time Series Analysis.

We used the regime shift analysis method (STARS) to determine the timing and magnitude of regime shifts (31). The regime shift index was calculated to measure the magnitude of the regime shift (*SI Appendix*, Materials and Methods). Significant changes in temporal trends of the time series were identified by the “segmented” package in the R environment (66) that indicates turning points of different evolution phases. We identified four statistically significant (*P* < 0.05) trend change point years at 2000, 1501, 709, and 544 CE (Fig. 3*B*). We used the Ensemble empirical mode decomposition (EEMD) method (67) to adaptively decompose the precipitation reconstruction to various climate components with different time scales. The DHL precipitation reconstruction was interpolated annually before performing the EEMD calculation.

### Comparison with Other Proxy Records and Simulation Data.

We compared our tree-ring δ^18^O precipitation reconstruction with other regional and global proxy records and simulation data (*SI Appendix*, Figs. S12–S15). This comparison with other proxies is constrained to general long-term trends (in some cases, even millennium timescales) rather than to multidecadal to centennial timescales, considering sampling resolution, depositional rate, and dating uncertainty in some proxy records; this includes the lower temporal resolution and uncertainty in timing of events inherent to radiocarbon or optically stimulated luminescence dating approaches.

## Supplementary Material

Supplementary File

## Data Availability

All data presented in this article have been deposited at the National Oceanic and Atmospheric Administration World Data Center Paleoclimatology Database (https://www.ncdc.noaa.gov/paleo/study/33654), and can be found in the online *SI Appendix*.
