# Supplementary material for: Long-term decrease in Asian monsoon rainfall and abrupt climate change events over the past 6,700 years
Source: Proc Natl Acad Sci U S A. 2021 Jul 19;118(30):e2102007118. doi: 10.1073/pnas.2102007118 (PMC8325342; doi:10.1073/pnas.2102007118)
Supplement: Supplementary File [file pnas.2102007118.sapp.pdf]

Supplementary Information for

Long-term decrease in Asian monsoon rainfall and abrupt climate change events over the past 6,700 years

Bao Yang, Chun Qin, Achim Bräuning, Timothy J. Osborn, Valerie Trouet, Fredrik Charpentier Ljungqvist, Jan Esper, Lea Schneider, Jussi Grießinger, Ulf Büntgen, Sergio Rossi, Guanghui Dong, Mi Yan, Liang Ning, Jianglin Wang, Xiaofeng Wang, Suming Wang, Jürg Luterbacher, Edward R. Cook, Nils Chr. Stenseth

Corresponding to: Nils Chr. Stenseth: n.c.stenseth@mn.uio.no; Bao Yang: yangbao@lzb.ac.cn

This PDF file includes:

Materials and Methods
Tables S1 to S8
Figs. S1 to S20
SI References (1–61)

Material and Methods

Study area and sample collection. The study area is situated at the northern fringe of the Asian summer monsoon (ASM) region, which is highly sensitive to climate variability (Fig. 1). According to meteorological records from Delingha national meteorological station (1956-2011), mean annual precipitation accounts to 170.4 mm, about 85% of which falls in summer (May-September). Mean air temperatures in summer and winter are 15.7°C and -9.4°C, respectively.

We collected on several occasions over the past decade wood samples from living and dead Qilian juniper (*Juniperus przewalskii* Kom.) trees in the Qilian Mountains (37–38.7°N, 97–100°E), northeastern Tibetan Plateau (TP). Qilian juniper is a dominant tree species growing in the cold and semi-arid conditions of the northeastern TP and is widely distributed on south-facing slopes (1, 2). We have produced a tree-ring width chronology that is currently the longest, absolutely dated tree-ring series for the northeastern TP spanning 4,649 years (2637 BCE–2011 CE) and representing moisture variations over the hydrological year (previous July to current June, or P7-C6) (1).

Logs of dead trees of the same species found recently at two sites close to the city of Delingha (Zongwulong Mountain: 37°27′N, 97°47′E, 3500-4150 m a.s.l.) represent a unique climatic archive, allowing the reconstruction of climate variability over the period from the mid-Holocene to the present.

The physiological mechanisms of the stable oxygen isotopic fractionation pathway from the source to the sink are clearly understood (3). By now, stable oxygen isotope variations ($\delta^{18}O$) in tree-ring cellulose are a frequently-used climate parameter in paleoclimate studies that serves as a reliable indicator of hydroclimatic conditions. In many cases, $\delta^{18}O$ series show stronger inter-tree correlations than ring-width series of the same trees. Hence, a lower number of trees is required to obtain a reliable chronological signal. Moreover, the analysis of stable isotopes can strongly improve the clarity of low-frequency climate signals, compared to the low-frequency signals stored in tree-ring widths (4).

In this study, 53 tree samples showing normal growth, clear ring boundaries and few missing rings were selected for $\delta^{18}O$ measurements. All 53 tree samples were collected from two open canopy sites within the Delingha region: MNT (37.45°N-37.46°N, 97.67°E-97.69°E) and QK (37.46°N-37.48°N, 97.77°E-97.78°E). The two sites represent two generally homogeneous growth environments in close proximity, located less than 30 km apart. The individual tree-ring samples were separated using a razor blade. For the period 1168-2011 CE, tree ring samples of 10 trees were pooled for the same calendar year, while the other 43 tree samples were measured individually tree by tree (see Table S1 for details). Precise dendrochronological crossdating allows us to pool samples and maximize the length of the record that can be produced with the available resources. Similarly, to reduce workload, $\delta^{18}O$ measurements were obtained at varying temporal resolutions (see Table S1 for details). Some samples were measured with 1-yr resolution in order to evaluate the impact of different resolutions on the chronology, such as MNP366A, MNP407B and MNP546A. The three samples do not start or end with "5" and "0". Some samples such as MNP367A, MNP364A, MNP405A, MNP344A, MNP375A, MNP533A, MNP387A are measured with 3-yr resolution, i.e. -182 BCE, -179 BCE, -176 BCE, ……, 1 CE, 4 CE, 7 CE, ……, 1948 CE. In addition, some samples such as MNT710B, ……, MNT265A have a 3-year resolution before 1800 CE and 1 year resolution for the period 1800 – 1955 CE over the recent millennium.

$\delta^{18}O$ analysis. The method of sample preparation and α-cellulose extraction followed standard laboratory guidelines as described by Wieloch et al. (5) and Qin et al. (6). For each tree sample, α-cellulose was extracted resulting in a total of 9526 samples for the measurements of $\delta^{18}O$. Of these, α-cellulose extraction for 6455 samples was undertaken in the isotope laboratory of the Institute of Geography (University of Erlangen-Nuremberg, Germany), and for 3071 samples in the key laboratory of Tree Ring and Environmental Change at Xishuangbanna Tropical Botanical Garden (Chinese Academy of Sciences, China). After freeze drying and sample homogenization, each α-cellulose sample (0.3-0.4 mg) was packed in silver capsules

for mass spectrometer analysis (Delta V Advantage, Thermo Electron, Bremen, Germany).

Isotopic compositions are presented as $\delta[‰]=(R_{sample}/R_{standard}-1)\times1000‰$, where R_{sample} and $R_{standard}$ are the $^{18}O/^{16}O$ ratios of the sample and the standard, respectively. The $^{18}O/^{16}O$ standard is the Vienna-Standard Mean Oceanic Water (VSMOW). A total of 9526 $\delta^{18}O$ values were obtained in this study, including 61 repeated measurements to assess for the differences in results obtained by the slightly different methods and laboratory standards used in the two abovementioned laboratories.

Multiple internal quality standard calibration for α-cellulose analysis is based on five laboratory standards in the German laboratory (IAEA 601, IAEA 602, a-Cellulose, Saccharose OAS, Stearic acid) and three laboratory standards in the Chinese laboratory (a-Cellulose, B2203 and B2205). The post-$\delta^{18}O$ measurement corrections included mean and drift correction. The standard deviation (SD) of the results from the repeated analysis of an internal standard (commercial cellulose) was better than 0.1‰ in both laboratories. In all analyses we used the arithmetic mean isotope ratios from different tree samples in the same calendar year.

Cross-laboratory validation showed no significant difference between the two sets of results (Fig. S1). Strong correlation between the chronologies measured by the pooled and individual α-cellulose extraction method is apparent, and no significant difference in mean $\delta^{18}O$ between the two approaches was detectable (Fig. S2).

δ^{18}O chronology establishment. Experiments and sensitivity tests were undertaken to investigate four potential non-climatic influences on the $\delta^{18}O$ measurements: sampling altitude, age-related trends, juvenile effects and outlier values.

Tree-ring $\delta^{18}O$ inherits the $\delta^{18}O$ signal of local precipitation via water absorption from the soil through the roots and may consequently contain an altitude signal. The trees sampled in this study grew at altitudes ranging between 3570 to 4085 m a.s.l.. More than half of the trees (28 trees) were located within a range of 100 m from 3895 to 3995 m a.s.l. (Fig. S3). For a sensitivity test, we compared two chronologies built from a) all 53 samples and b) from 39 samples within the narrow altitudinal range from 3850 to

4085 m a.s.l. and only found little differences. As a further test, we investigated whether a relationship might be present between mean $\delta^{18}O$ and mean elevation of the samples. Their time series showed different patterns over the past 6700 years (Fig. S3), and this was verified by scatter plots of the $\delta^{18}O$ values and elevation over the entire period (Fig. S3), as well as during the instrumental period (Fig. S4). In no case a significant relationship between mean $\delta^{18}O$ and mean elevation of the samples was detectable.

The same lack of influence of altitude on $\delta^{18}O$ can be observed for a $\delta^{18}O$ series from the Animaqin mountains (34.76°N, 99.69°E, northeastern TP, approximately 300 km from our study site). In this recent study, potential effects of altitude and juvenile growth stages on tree-ring cellulose $\delta^{18}O$ in Qilian junipers were assessed (7). It was revealed that tree-ring $\delta^{18}O$ data from five different altitudes (4250, 4150, 4000, 3900 and 3800 m a.s.l.) were strongly correlated ($r > 0.88$) and no significant differences were detected in the mean or SD of tree-ring $\delta^{18}O$ values at different elevations. Therefore, altitude effects on tree-ring $\delta^{18}O$ were not observed in that study area, which confirmed our finding. In addition, Wang et al. (8) developed a 3-year-resolution $\delta^{18}O$ chronology spanning 991-2010 CE based on 20 living Qilian juniper trees in the same region (37°16′N, 97°32′E) as our study. All tree samples were collected from the same altitude of 4000 m a.s.l., which is higher than most tree stands in our study. However, the average $\delta^{18}O$ value (32.9‰) is comparable to our study (32.7‰) over the same period 991-2010 CE. Therefore, a possible elevation effect/dependency can be excluded.

Age-related effects on tree-ring $\delta^{18}O$ values have been proposed for junipers in Pakistan (9), but similar results were not found consistently in Asia. Hence, no consensus on age-dependent effects has been reached (10). An effect of age on tree-ring cellulose $\delta^{18}O$ was limited to the first 10 years of tree growth in juvenile Qilian junipers in the Animaqin Mountains of the northeastern TP (7), but not found in other studies focusing on $\delta^{18}O$ in juniper trees from the southern TP (11). In addition, age effects were not detected in pines from south-east China or in larch trees from Bhutan (12, 13).

In our study, 36 of the 53 samples contained full piths. We adopted linear fitting to the time series of these 36 samples to detect any age effect in each $\delta^{18}O$ series, on three time scales: 1) the whole series; 2) the first 50 years of tree growth; and 3) ages beyond 50 years (Fig. S5, S6, Table S2). Over the whole series, 7 out of 36 series showed significantly positive trends, 17 out of 36 series showed significantly negative trends, and 12 out of 36 series showed no significant trends. Over the first 50 years of tree growth, 2 out of 36 series showed significantly positive trends, and 34 out of 36 series showed no significant trends. At ages beyond 50 years, 9 out of 36 series showed significantly positive trends, 15 out of 36 series showed significantly negative trends, and 12 out of 36 series showed no significant trends. In summary, there was no consistent relationship between tree age and $\delta^{18}O$, whether considering the whole series, the first 50 years of growth, or the remainder. Therefore, we conclude that the $\delta^{18}O$ series contain no age-related trend and can therefore seamlessly be merged into a mean series.

We also carried out a sensitivity analysis to assess a potential juvenile effect, by comparing the arithmetic means of each individual $\delta^{18}O$ series after removing the first 50 or 100 years. The resulting $\delta^{18}O$ chronologies were very strongly correlated with the original $\delta^{18}O$ chronology ($r>0.98$, $n=1628$, 4405 BCE to 2011 CE, $P<0.01$), and there was no significant difference in their mean values ($p<0.01$) (Fig. S6), suggesting there is no juvenile effect on tree-ring cellulose $\delta^{18}O$ in these trees. Therefore, local age-related influences on tree-ring cellulose $\delta^{18}O$ in the northeastern TP are negligible. Similarly, the arithmetic mean of each $\delta^{18}O$ series is not affected by altitude.

The outliers detected in our tree-ring $\delta^{18}O$ series probably originate from cellulose impurities or from contamination during the processes of α-cellulose extraction, cellulose packaging and measurement. Firstly, 34 outliers were detected on a tree-by-tree basis using the Pauta criterion (mathematical expectation ±3×SD as the threshold of rejection level). Next, the same screening method was applied to non-outlier measurements to re-establish a credible interval for each calendar year, before assessing whether the above outliers were reasonable. Twelve outliers were not excluded based on the above analyses (Table S3). Meanwhile, four apparent outliers (37.08‰, 35.34‰,

35.91‰ and 36.69‰) in 1950 BCE showed consistent positive anomalies of 4.64‰, 4.37‰, 5.79‰ and 4.68‰; these were similar to the other non-outlier measurements (35.46‰, 35.26‰) with positive respective anomalies of 4.27‰ and 3.44‰. Such values may indicate a common climate imprint. Therefore, the four outliers in 1950 BCE were not excluded. Five additional outliers in the pooled series “MNT” were detected (excluding the abovementioned outlier in 1192 CE, Table S3). The MNT outliers in 1354 CE (27.52% with -5.04% bias from five trees) were consistent with other non-outlier measurements (31.59‰ with -1.51‰ bias and 30.96‰ with -1.76‰ bias) and the outliers in 1615 CE (27.93‰ with -4.63‰ bias from ten trees) were consistent with other non-outlier measurements (29.25‰ with -3.24‰ bias and 29.83‰ with -2.89‰ bias). The MNT outliers (36.92‰ from ten trees) in 1810 CE were retained because there were no other measurements in that year. The other outliers (37.37‰ in 1171 CE and 37.82‰ in 1183 CE, both from one tree) were excluded. Overall, 16 outliers were excluded and 18 apparent outliers were retained (Table S3).After completing the above analyses, a merged $\delta^{18}O$ chronology was developed based on the arithmetic mean of all the $\delta^{18}O$ series for the same calendar years. The final chronology has at least one sample per measured calendar year (either one year in three or one year in five, annually since 1951) from 4680 BCE to present, replication is at least two samples for most of this period (4510 BCE to present), though for most of the record sample depth ranges from four up to twelve individuals. The long-term arithmetic mean isotope ratio was 31.88‰.

As indicated above, $\delta^{18}O$ measurements were obtained at varying temporal resolutions over time. We compared the merged 5-year resolution $\delta^{18}O$ chronology with time series of three individual $\delta^{18}O$ records that have annual resolution during 2550 BCE–1100 CE (Fig. S8). The individual annual $\delta^{18}O$ records generally track the direction of the DLH mean $\delta^{18}O$ chronology with 5-year resolution. This agreement suggests that the composite chronology is insensitive to individual $\delta^{18}O$ records with different temporal resolutions.

Sample size, mean interseries correlations (Rbar) and Expressed Population Signal (EPS) of the $\delta^{18}O$ dataset. The calculation is complicated because some series are measured only every 5th ring, some only every 3rd ring, some every ring, and one series is based on pooled material from multiple trees. For year X, sample size is the count of how many series have at least one measurement during the period X-2 to X+2. This is to avoid large fluctuations in EPS from year to year when the series with a measurement every 5th year is present. But it must be interpreted carefully: when calculated this way, the EPS has values every year but they only represent the quality of the chronology for the years with data. If the pooled series has data in the X-2 to X+2 period, the sample size includes all of the constituent trees used in the pooled series in year X.

A window of 250-year centered on year X is used (from year X-124 to X+125) to calculate correlation and Rbar. All series with data during this window are considered on a pairwise basis. The correlation between each pair of measurement series is calculated using only the values in the window, but is only calculated if there is a minimum of 10 values (i.e. both series must have measurements from the same year for at least 10 years during the window). This minimum overlap requirement is to avoid correlations being computed from very short overlaps because they would have large uncertainty.

By the above process, a correlation matrix is generated for each window. If the correlation matrix contains at least 3 correlations (i.e. at least 3 pairs of measurement series met the above criteria) then it is averaged to obtain Rbar for this window. For years when the pooled series has data but there are not sufficient other series to calculate Rbar (using the above criteria), then the mean Rbar for the whole chronology is used. EPS is calculated from the sample size and Rbar using the standard formula of Wigley et al. (14).

Hydroclimate reconstruction. We obtained monthly instrumental records (precipitation, temperature, and relative humidity) of Delingha during 1956-2011 CE from the National Meteorological Information Centre of China. The outlier of monthly

precipitation (137.1 mm) in August, 1977 was adjusted to 22.1 mm according to records of the surrounding meteorological stations (15). The self-calibrating Palmer Drought Severity Index (scPDSI) data is the version of CRU 1901-2017 0.5° Global 3.26 available from: https://crudata.uea.ac.uk/cru/data/drought/#global.

As mentioned above, there is a significant correlation and no significant difference ($p<0.05$) in the mean values between the $\delta^{18}O$ chronologies established by individual and pooling methods. Considering that the whole chronology is mainly established by individual $\delta^{18}O$ series except for the past 1168 years, we adopted the arithmetic mean of the individual $\delta^{18}O$ series as the chronology for climate calibration. Correlation and response analysis of $\delta^{18}O$ to climatic factors was conducted over the period 1956–2011 (Fig. S9). Our record shows a strong correlation between $\delta^{18}O$ and precipitation of the hydrological year (prior August to current July; $r_{1956\text{-}2011} = -0.70$； first-order difference correlation=-0.62), April-September scPDSI ($r_{1956\text{-}2011} = -0.69$； first-order difference correlation=-0.62), and May-September relative humidity (r=-0.48, first-order difference correlation=-0.58). As indicated in Tables S4–S5, our tree-ring $\delta^{18}O$ series serves as a strong indicator of interannual variability in drought conditions and precipitation.

Calibration against the annual precipitation record allows us to quantify hydroclimatic variability on multi-annual to orbital timescales. Ordinary regression analysis showed that regression residuals were significantly correlated (lag-1 autocorrelation = 0.38, $p<0.01$) over time, violating the assumption that the errors are independent of each other. We adopted a first-order autoregressive model for the random error accounting for the autocorrelation of the errors using the AUTOREG procedure in SAS 9.4 (SAS Institute Inc., Cary, NC). The relationship between annual precipitation and tree-ring oxygen isotopes was analyzed and the estimated coefficients of the model were used to reconstruct the annual (prior August to current July) precipitation of the past 6700 years. The estimates of the regression coefficients and autoregressive parameters are confident at the 0.01 significance level. The annual precipitation reconstruction explains 49% (n=56, $p<0.01$) of the variance in the Delingha instrumental precipitation record.

We use a "leave-one-out" cross-validation to evaluate the statistical skill of our reconstruction model. For small sample sizes (56 annual values here; 1956-2011 CE), this approach is preferable to splitting the available data into separate calibration and validation sets. The procedure runs the AUTOREG model each time by leaving one observation out, thus creating a new dataset with the observations and the predictions (each prediction is one-datum validation). The cross-validation is assessed using the Reduction of Error (RE). The RE is a powerful test statistic, penalizing errors in the amplitude (from the mean level of the calibration period) of predicted values (1). The mean RE is 0.46, demonstrating the validity of our reconstruction model.

The uncertainty ranges for the average precipitation of some sub-periods of the entire reconstruction series are calculated with a modification factor multiplying the ± 1 RMSE (root mean square error) based on the method used by Briffa et al. (16), since uncertainty ranges are timescale-dependent.

Model description and simulation experiments. A set of transient simulation experiments (TraCE-21ka, Simulation of Transient Climate Evolution over the past 21 000 years) conducted with the Community Climate System model version 3 (CCSM3) was used to reveal spatial representation of the Delingha precipitation and for comparison with our tree-ring $\delta^{18}O$ record. The orbital-induced solar insolation, atmospheric greenhouse gas concentrations, meltwater fluxes, and continental ice sheets were represented in the TraCE-21 transient simulation (17). The experiments consist of one transient experiment with all forcings (TraCE-ALL), one single-forcing experiment forced only by transient orbital variation (TraCE-ORB), one single-forcing experiment forced only by transient meltwater flux (TraCE-MWF), one single-forcing experiment forced only by a quasi-transient ice sheet (TraCE-ICE), and one single-forcing experiment forced only by transient greenhouse gas concentration changes (TraCE-GHG). The simulations were conducted from 20 000 BCE to 1990 CE for the TraCE-ALL, the TraCE-ORB and the TraCE-GHG experiments, and from 17 000 BCE to 1990 CE for the TraCE-MWF and the TraCE-ICE experiments (see ref Yan and Liu (18) for details). It should be noted, that according to the TraCE-ALL and TraCE-MWF

experiments no freshwater was delivered to the ocean after 3000 BCE. Detailed information about the experiment design can be found in refs He (19) and He et al. (20). The model has been used to investigate the causes and mechanisms of Holocene climate changes, such as the Bølling–Allerød warming (17), the cooling into the Younger Dryas, and the following recovery to warm conditions (21), as well as the El Niño–Southern Oscillation (ENSO) evolution over the past 21 kyr (22). In the present work, we chose the period covering the past 6700 years.

In addition, simulation dataset comprising 13 parallel experiments from the global Community Earth System Model-Last Millennium Ensemble (CESM-LME) are used as a supplement of the TraCE-21ka simulation. The simulation is conducted from 850 to 1850 CE and possessed ~2-degree resolution for the atmosphere and land components and ~1-degree resolution in the ocean and sea ice components (23). The CESM-LME includes a group of more comprehensive external forcings and its ensemble results are less dependent on the initial conditions and the contingency of the model simulation than a single member simulation, and therefore is more credible than other models that participate in the Coupled Model Intercomparison Project Phase 5 (CMIP5). The forcings include the transient evolution of solar activity, volcanic emissions, greenhouse gases, aerosols, land use conditions, and orbital parameters. We conducted the Empirical Orthogonal Function (EOF) analysis based on the TRACE-21ka (over the Holocene) and CESM-LME (850-1850 CE) simulation results.

Regime shift analysis (STARS) and change points analysis of temporal trends of the time series. The regime shift analysis (STARS) is based on a sequential t-test approach to determine the timing of regime shifts (24). It calculates a Regime Shift Index (RSI), which represents a cumulative sum of normalized deviations from the hypothetical mean level for the new regime. The magnitude of RSI is a measure of the magnitude of the shift. The parametric method requires a user to set a number of parameters, such as the target significance level, cut-off length, and Huber weight parameter. The highest RSIs, representing the most dramatic decreases in mean annual precipitation level and thus the strongest shifts towards drought conditions were

detected in 3350, 2815, 2095, 1675, and 70 BCE (Table S7). Shifts towards wetter conditions were typically less dramatic, with lower absolute RSI values, but the strongest wet regime shifts occurred in 2565 and 1185 BCE and ~760 CE (Table S7). The high-resolution dating of these regime shifts, allows us to determine the duration and magnitude of past drought events and pluvials. A longer cut-off length yields a stronger signal. STARS is available online at http://www.beringclimate.noaa.gov/regimes (24). Significant changes in temporal trends of the time series were identified by the “segmented” package in the R environment (25, 26), that indicate turning points of different evolution phases. We identified four statistically significant (p < 0.05) trend change point years – at 2000 BCE, 1501 BCE, 709 BCE, and 544 CE – that indicate turning points of different evolution phases.

Comparison with other proxy records and simulation data. We compared our $\delta^{18}O$ chronology with other regional as well as global proxy records and model simulation data. Other proxy evidence includes stalagmite $\delta^{18}O$ records, sediment section records derived from closed lakes, aeolian-sand and loess–paleosol sections, tree pollen percentages, pollen-based precipitation reconstructions and temperature reconstructions, and archaeological data (Refs 27–58, Tables S6, Figs. S12–S15, S19-S20). Our tree-ring $\delta^{18}O$ series is significantly correlated with the TRW chronology and ice-core $\delta^{18}O$ record on a decadal timescale during the common time period between 990-1980 CE (Fig. S12). We also compared our $\delta^{18}O$ chronology and a $\delta^{13}C$ record of buried wood fragments (*Picea crassifolia* Kom.) from the Qinghai Lake basin (27). Lu et al. (27) provided radiocarbon dates of Qinghai spruce woody debris in tandem with $\delta^{13}C$ data. The $\delta^{13}C$ of each wood sample is a valuable addition to our hydroclimatic reconstruction, enabling a further examination of the Holocene climatic trends. Tree-ring $\delta^{13}C$ correlates significantly with PDSI over the instrumental period and is thus a good indicator of drought variability (59). Liu et al. (59) developed Qinghai spruce $\delta^{13}C$ chronologies at five sites in the Qilian Mountains, at elevations of 2440-3360 m. Here, we chose the Sidalong site chronology for comparison because its altitude of 3360 m

a.s.l. is close and comparable to that of the wood remains collected by Lu et al. (27) (3482 m a.s.l.). We used the $\delta^{13}C$ mean over the period 1891-2003 AD to represent the $\delta^{13}C$ for 1950 AD as a comparison. Thus, an intermittent $\delta^{13}C$ curve comprising $\delta^{13}C$ data for the past and the present can be plotted (Fig. S13). Some key inferences can be made based on the general trend represented by the $\delta^{13}C$ curve, even if the means of tree-ring $\delta^{13}C$ values are inevitably influenced by potential juvenile and altitude effects, as well as different sampling heights, directions, and areas (stem or branches). A long-term aridification trend is apparent in the $\delta^{13}C$ series from roughly 5000 to 2200 BCE and comparable to the trend in our $\delta^{18}O$-based reconstruction (Fig. S13), lending robust support to our interpretation. The climatic or environmental implications of each proxy record have been interpreted and reviewed in detail (60). Comparison with other proxy records is constrained to general long-term trends.

Tables (S1-S8)

Table S1. Statistics for the 53 Qilian juniper samples (39 dead trees, 14 living trees) used for $\delta^{18}O$ analysis. Living trees are those with an end year of 2010 or later. Samples are divided into annually-resolved subsamples. Subsequently, subsamples were analyzed for their $\delta^{18}O$ values at intervals varying from 1 to 5 years as indicated by the resolution. For the period 1168-2011 CE, tree ring samples of 10 trees were pooled for the same calendar year, while the other 43 tree samples were measured individually tree by tree. Trees marked with * were used in the pooled analysis (subsamples for the same calendar year but from different trees were pooled before $\delta^{18}O$ measurement), while those marked ** were used for climate calibration.

Code	Latitude	Longitude	Start year	End year	Resolution (Year)	Elevation (m.a.s.l)	Minimum TRW (mm)	Mean TRW (mm)	Maximum TRW (mm)
DQP680A	37.47	97.77	-4680	-3800	5	3998	0.04	0.16	0.46
5QP171A	37.47	97.77	-4505	-4140	5	3897	0.06	0.31	0.63
5QK191D	37.47	97.77	-4180	-3200	5	3882	0.01	0.12	0.27
DQP502A	37.47	97.77	-4095	-3120	5	3921	0.03	0.24	0.69
DQP324A	37.48	97.78	-3985	-3010	5	3989	0.04	0.18	0.64
DQP242A	37.47	97.78	-3350	-2535	5	3955	0.01	0.28	0.97
DQP226B	37.48	97.77	-3110	-2420	5	4002	0.04	0.22	0.66
DQP323A	37.48	97.78	-2930	-2270	5	3986	0.01	0.23	0.60
DQP201A	37.47	97.77	-2815	-2320	5	4048	0.01	0.21	0.65
DQP243A	37.47	97.78	-2810	-1850	5	3959	0.01	0.23	0.64
DQP103A	37.46	97.77	-2720	-1795	5	3853	0.01	0.27	0.61

MNP546A	37.46	97.67	-2558	-1638	1	3950	0.01	0.24	0.66
MNP946A	37.46	97.66	-2515	-1730	5	3841	0.04	0.22	0.71
MNP366A	37.45	97.68	-2284	-1087	1	3995	0.01	0.17	0.58
MNP407B	37.45	97.68	-2080	-1505	1	3951	0.01	0.20	0.54
MNP311A	37.45	97.68	-1705	-1100	5	3962	0.01	0.30	0.77
MNP372A	37.45	97.68	-1685	-1055	5	3975	0.03	0.22	0.58
MNP324A	37.45	97.68	-1620	10	5	3973	0.01	0.20	0.61
MNP376A	37.45	97.68	-1610	-1095	5	3975	0.01	0.22	0.53
MNP312A	37.45	97.68	-1470	-1095	5	3962	0.01	0.21	0.64
MNP545A	37.45	97.67	-1455	585	5	3935	0.01	0.24	0.85
DQP111A	37.47	97.77	-1215	-65	5	3897	0.01	0.34	1.06
DQP207A	37.47	97.77	-1175	-360	5	4085	0.05	0.25	0.62
MNP988A	37.46	97.66	-1095	75	5	4084	0.01	0.23	0.53
MNP542A	37.46	97.68	-840	-125	5	3960	0.01	0.25	0.61
MNP400B	37.45	97.68	-440	65	5	3944	0.01	0.42	0.89
MNP240A	37.45	97.68	-405	115	5	3855	0.01	0.41	1.06
MNP367A	37.45	97.68	-182	1588	3	3985	0.03	0.25	0.69
MNP265A	37.45	97.68	-150	355	5	3967	0.01	0.31	0.70
MNP364A	37.45	97.68	-101	691	3	4019	0.03	0.25	0.99
MNP405A	37.45	97.68	-32	1012	3	3965	0.01	0.23	0.71
MNP344A	37.45	97.68	-20	1376	3	3981	0.01	0.31	0.87
MNP375A	37.45	97.68	13	595	3	3975	0.01	0.20	0.68
MNP533A	37.45	97.68	34	1624	3	3937	0.01	0.26	0.92

MNP387A	37.45	97.68	628	1621	3	3960	0.01	0.24	1.05
MNT710B*	37.45	97.68	1167	1910	1,3	3885	0.01	0.36	1.41
MNT380A*	37.45	97.68	1291	2011	1,3	3780	0.01	0.28	0.94
MNT589A*	37.45	97.68	1312	2011	1,3	3764	0.06	0.41	0.92
MNT313A*	37.45	97.68	1333	2010	1,3	3825	0.03	0.41	1.08
MNT038A*	37.45	97.68	1344	1943	1,3	3798	0.01	0.29	1.01
MNT585B*	37.46	97.67	1384	1903	1,3	3975	0.01	0.38	1.20
MNT360B*	37.45	97.68	1396	2011	1,3	3745	0.01	0.34	0.90
MNT304A*	37.45	97.68	1438	2010	1,3	3800	0.03	0.46	1.33
MNT261A*	37.45	97.68	1462	1879	1,3	3870	0.01	0.31	0.87
MNT265A*	37.45	97.68	1574	2010	1,3	3970	0.03	0.50	1.42
QKC103A**	37.46	97.77	1894	2017	1	3848	0.29	0.74	1.63
MNT611B**	37.45	97.67	1900	2011	1	3785	0.04	0.44	1.42
DQC206A**	37.47	97.77	1900	2012	1	3923	0.01	0.41	1.00
MNT619A**	37.45	97.67	1951	2011	1	3790	0.26	0.41	0.56
MNT629A**	37.45	97.69	1951	2011	1	3570	0.06	0.35	0.69
MNT631A**	37.45	97.69	1952	2011	1	3570	0.10	0.79	1.25
MNT618A**	37.45	97.67	1953	2011	1	3790	0.18	0.51	0.75
MNT598A**	37.45	97.68	1964	2011	1	3760	0.34	0.55	0.74

Table S2. Statistics for the trend significance test based on linear fitting to each $\delta^{18}O$ series. We assume that all series started from the first cambial year (see Fig. S5 for further details). Headings: “+” indicates the slope is positive and significant at the 95% confidence level, “-” indicates the slope is negative and significant at the 95% confidence level, “/” indicates there is no significant trend at the 95% confidence level.

Biological Age	+	/	-
1-50a	2	34	0
>51a	9	12	15
The whole series	7	12	17

Table S3. Results of outlier analyses for the 44 $\delta^{18}O$ series.

Year	Retained outlier (‰)	Mean±SD(‰), series length*	Mean±SD(‰), sample depth**	Year	Excluded outlier (‰)	Mean±SD(‰), series length*	Mean±SD(‰), sample depth**
1950	37.08	34.57±1.56, 118	35.36±0.10, 2	-4420	35.89	29.13±1.34, 171	31.37, 1
1950	35.91	30.12±1.61, 158	35.36±0.10, 2	-3495	38.46	29.86±1.47, 188	28.02±0.34, 2
-950	36.69	32.01±1.47, 316	35.36±0.10, 2	-3350	38.89	31.35±1.60, 164	30.85±1.65, 2
**1950**	**35.34**	30.96±1.29, 775	35.36±0.10, 2	-2268	26.67	31.82±1.45, 777	29.09, 1
**1949**	**35.21**	30.96±1.29, 775	35.32±0.26, 2	-1852	25.90	31.82±1.45, 777	30.12, 1
**1820**	**26.99**	32.01±1.47, 316	28.88±0.96, 3	-1642	27.01	30.96±1.29, 775	-
**1650**	**36.78**	31.82±1.45, 777	32.71±2.05, 3	-1400	25.59	31.62±1.30, 320	33.41±1.65, 6
**1495**	**36.40**	31.82±1.45, 777	33.75±1.54, 4	-410	22.92	31.25±2.08, 219	32.59±1.47, 6
**1455**	**38.69**	31.82±1.45, 777	35.67±1.80, 4	-116	28.27	33.10±1.60, 572	-
**1425**	**36.73**	31.82±1.45, 777	34.02±2.21, 4	-113	28.21	33.10±1.60, 572	-
**-375**	**26.50**	31.94±1.73, 140	30.49±1.70, 7	-106	28.30	33.10±1.60, 572	-
**661**	**37.59**	32.72±1.37, 327	36.63±0.76, 4	118	38.65	32.49±1.60, 481	35.24±1.07, 4
**1192**	**37.34**	32.56±1.45, 413	33.69±1.35, 3	515	26.34	31.60±1.37, 321	28.65, 1
1354	27.52	32.56±1.45, 413	31.28±0.32, 2	784	38.22	33.17±1.52, 451	34.09±1.29, 4
1615	27.93	32.56±1.45, 413	29.54±0.29, 2	1171	37.37	32.56±1.45, 413	32.98±1.22, 4
1810	36.92	32.56±1.45, 413	-	1183	37.82	32.56±1.45, 413	32.86±1.15, 4
**1956**	**37.25**	33.12±1.28, 112	34.99±1.94, 7				

**2005**	**28.41**	33.12±1.28, 112	30.42±1.11, 8

Note: Retained outliers (abbrev., Retained), Excluded outliers (Excluded), the corresponding mean value (Mean) ± the standard deviation (SD) and the series length (sample depth) listed in the table. Outlier analysis was applied to each $\delta^{18}O$ series based on the Pauta criterion (mathematical expectation±3*SD as the threshold of rejection level, the corresponding Mean±SD and the series length were denoted as *), and 34 outliers were identified. Subsequently, the same screening method was applied to each calendar year (the corresponding Mean±SD and the sample depth were denoted as **), and 12 outliers (bold) were retained. 6 outliers, explained by other records (normal) or unique values (gray) in the left panel, were also retained. In total, 18 outliers were retained, and 16 outliers were excluded. Years with prefix symbol "-" mean BCE, and the others mean CE.

T**able S4** Maximum correlation between δ^{18}O chronology and various seasonal climatic parameters (1956–2011).

Signal	Seasonal assemblage	Correlatio n	First-order difference correlation
Temperature	C1-9	-0.18	-0.17
Precipitation	P8-C7	**-0.70**	**-0.62**
scPDSI	C4-9	**-0.62**	**-0.62**
Relative humidity	C5-9	**-0.48**	**-0.58**

Bold values indicate that the correlation is significant at $p < 0.01$.

Table S5. Statistics of the split-period calibration and verification tests (1956–2011).

	Calibration (1956–1991)	Verification (1992–2011)	Calibration (1976–2011)	Verification (1956–1975)	Full Calibration (1956– 2011)
R	0.68	0.65	0.66	0.48	0.70
R^2	0.46	0.42	0.44	0.23	0.48
RE	-	0.55	-	0.65	0.46
CE	-	0.41	-	0.21	-
Sign test	25+/11-[a]	17+/3-[b]	25+/11-[a]	18+/2-[b]	-

Re: Reduction of Error, CE: Coefficient of Efficiency.

a Significant at $p < 0.05$. b Significant at $p < 0.01$.

Table S6. Locations of Holocene paleoclimatic records used for comparison in this study.

Site No.	Site name	Lat.(°N)	Long.(°E)	Dating material	Dating methods	Numbers of dates	Dating error	Temporal resolution (Year)	References
1	Jiuxian Cave	33.57	109.1	Speleothem	U/Th	9961(22), 9962 (9)	9961 (368.64), 9962 (542.22)	9961 (8.4), 9962 (40.13)	Cai et al. (2010)
2	Xianglong Cave	33	106.33	Speleothem	U/Th	XL2 (19), XL16 (23), XL26 (15)	XL2 (14), XL16 (15.87), XL26 (20.93)	XL2 (10.22), XL16 (10.86), XL26 (9.88)	Tan et al. (2017)
3	Heshang Cave	30.45	110.42	Speleothem	U/Th	15	60.07	6.23	Hu et al. (2008)
4	Dongge Cave	25.28	108.08	Speleothem	U/Th	YDX 2004 (10), WYJ2004 (38), Dykoski 2005 (21)	YDX 2004 (48.3), WYJ2005 (59.42), Dykoski 2005 (57.81)	YDX 2004 (79.8), WYJ2005 (4.22), Dykoski 2005 (18.25)	Yuan et al. (2004), Wang et al. (2005), Dykoski et al. (2005)
5	Sanbao Cave	31.67	110.43	Speleothem	U/Th	SB10 (5), SB27 (6), SB26 (5), SB43 (11)	SB10 (36.8), SB27 (81.67), SB26 (44.2), SB43 (47.91)	SB10 (112.39), SB27 (40.22), SB26 (18.49), SB43 (30.16)	Dong et al. (2010)
6	Lianhua Cave	29.48	109.53	Speleothem	U/Th	27	144	10.4	Dong et al. (2018)
7	Qunf Cave	17.17	54.3	Speleothem	U/Th	11	117.36	7.23	Fleitmann et al. (2003)
8	Sihailonwan Maar Lake	42.28	126.6	Bulk organic matter	AMS	19	40.68	60.23	Stebich et al. (2015)
9	Bayanchagan Lake	41.65	115.21	Bulk organic matter	AMS	4	57.5	208.34	Jiang et al. (2006)
10	Diaojiaohaizi Lake	41.3	112.35	Bulk organic matter	14C	2	85	129.42	Song et al. (1996)

11	Daihai Lake	40.55	112.66	Bulk organic matter	AMS	6	30.33	90.26<br>29.56	Xiao et al. (2004)<br>Xu et al. (2010)
12	Qinghai Lake	36.67	100.51	Bulk organic matter	AMS	5	110	38.69	Shen et al. (2005)
13	Dalianhai Lake	36.25	100.41	Bulk organic matter	AMS	3	53	76.25	Cheng et al. (2013)
14	Gonghai Lake	38.9	112.23	Bulk organic matter	14C	14	32.57	13.31	Cheng et al. (2015a)
15	Animaqing Mountains	35.0	100.07	Tree ring	Cross-dating	NA	NA	NA	Xu et al. (2017)
16	Yaoxian section	34.53	108.45	Quartz grains	OSL	5	184	51.21	Zhao et al. (2007), Xia et al. (2014)
17	Baijian Lake	39.06	104.15	Quartz grains	OSL	16	193.75	338.95	Long et al. (2012)
18	Xingyun Lake	24.34	102.8	Bulk organic matter	AMS	5	66.2	188.77	Chen et al. (2014)
19	Dali Lake	43.15	116.29	Shell and Charcoal	14C, OSL	9	255.88	NA	Goldsmith et al. (2017)
20	Xinjie site	31.37	119.7	Total organic carbon	AMS, 14C	6	76	81.35	Lu et al. (2018)
21	Northern Monsoon Margin	35-50	96-130	N/A	N/A	N/A	N/A	194	Wang and Feng (2013)
22	Monsoonal eastern China	20-52	110-135	N/A	N/A	N/A	N/A	100	Wang et al. (2010)
23	Northern China sandlands	37.27-49.58	107.20-124.50	Quartz grains	OSL	89 dates at 44 sites	NA	200	Wang et al. (2014)
24	Yulin section	38.33	109.73	Quartz grains	OSL	3	NA	268.35	Lu et al. (2013)
25	Chinese Loess Plateau	34-40	103-114	Quartz grains, Bulk organic matter	TL, OSL, 14C	1 TL date, 77 OSL dates, 181 ^{14}C dates	NA	200	Wang et al. (2014), Chen et al. (2015a)

26	Cariaco Basin Ti concentrations	10.7	-65.1	Planktic foraminifer *Globigerina bulloides*	AMS	10	N/A	4-5	Haug et al. (2011)

Table S7. Results of the regime shift analysis (STARS) to detect shifts in the mean of the reconstructed annual precipitation. To identify the years of major change, a large cut–off length of 50 data points was chosen, corresponding to a period of 150-250 years (depending on sampling resolution; see text for details) in the DLH $\delta^{18}O$ chronology. We used a probability level equal to 0.01 and Huber parameter equal to 1.

Year of shift	Conf	RSI	Sub-period	Length (yr)	Annual precipitation Mean (mm)
			4680-4290 BCE	395	297.62±11.33
4285 BCE	$< 10^{-4}$	0.12	4285-3605 BCE	685	272.94±8.60
3600 BCE	$< 10^{-6}$	-0.14	3600-3355 BCE	250	299.71±14.24
3350 BCE	$< 10^{-11}$	0.36	3350-2820 BCE	535	252.77±9.74
2815 BCE	$< 10^{-6}$	0.34	2815-2570 BCE	250	207.03±14.24
2565 BCE	$< 10^{-5}$	-0.25	2565-2100 BCE	470	246.63±10.39
2095 BCE	$< 10^{-9}$	0.37	2095-1680 BCE	420	205.86±10.99
1675 BCE	$< 10^{-9}$	0.38	1675-1190 BCE	490	161.93±10.17
1185 BCE	$< 10^{-10}$	-0.28	1185-75 BCE	1115	200.12±6.74
70 BCE	$< 10^{-10}$	0.34	70 BCE to 343 CE	413	164.69±8.83
346 CE	$< 10^{-11}$	0.20	346-760 CE	417	124.62±8.54
763 CE	$< 10^{-14}$	-0.19	763-1342 CE	582	162.63±7.23
1345 CE	$< 10^{-6}$	-0.11	1345-1447 CE	105	198.13±17.02
1450 CE	$< 10^{-5}$	0.09	1450-2002 CE	555	163.86±6.77
2005 CE	0.26	-0.42	2005-2011 CE	7	230.55±38.06

Note: RSI, Regime Shift Index; Mean, Equal-weighed arithmetic means of the regimes; Length, Length of the regimes; Difference, the difference between the mean values of the neighboring regimes; Conf, Confidence level of the difference between the mean values of the neighboring regimes based on the Student's two-tailed t-test with unequal variance (TTEST procedure in Excel).

Table S8. Absolute dry and wet extreme years over the past 6700 years. In total, 157 drought and 164 humid events were identified. Extremes in the precipitation reconstruction were assigned as years in which the precipitation exceeded the whole-period 10th and 90th percentiles.

Dry and wet extreme years							
dry	wet	dry	wet	dry	wet	dry	wet
-2700	-4670	223	-4315	604	-3670	1006	-3200
-2570	-4665	226	-4310	613	-3660	1009	-3185
-1950	-4660	250	-4300	616	-3655	1048	-3165
-1675	-4655	253	-4295	628	-3615	1132	-3145
-1625	-4640	256	-4290	631	-3600	1165	-3140
-1565	-4635	262	-4255	634	-3590	1192	-3135
-1540	-4630	265	-4245	643	-3580	1240	-3100
-1515	-4625	283	-4210	646	-3550	1255	-3080
-1500	-4620	286	-4205	658	-3540	1261	-3025
-1495	-4615	289	-4140	661	-3535	1276	-3015
-1455	-4605	349	-4135	664	-3520	1480	-3010
-1425	-4590	358	-4130	667	-3515	1486	-3000
-1410	-4585	361	-4125	673	-3510	1498	-2970
-1395	-4580	364	-4120	679	-3500	1516	-2940
-1380	-4575	382	-4115	685	-3495	1549	-2895
-1365	-4570	388	-4080	691	-3490	1552	-2885
-1340	-4565	394	-4045	703	-3485	1555	-2875
-1215	-4560	397	-4000	706	-3470	1585	-2855
-1210	-4555	400	-3990	709	-3465	1627	-2825
-1205	-4550	415	-3975	712	-3460	1633	-2585
-1145	-4545	427	-3940	715	-3450	1783	-2555
-1050	-4535	439	-3910	721	-3445	1789	-2540
-940	-4530	448	-3890	724	-3435	1810	-2535
-920	-4520	454	-3885	736	-3425	1831	-2500
-655	-4515	457	-3865	742	-3420	1843	-2455
-590	-4510	463	-3825	745	-3410	1876	-2450
-440	-4405	478	-3815	760	-3405	1894	-2440
-350	-4400	484	-3805	787	-3395	1909	-2415
-260	-4395	523	-3800	790	-3390	1918	-2405
-70	-4390	526	-3785	814	-3385	1930	-2335
-20	-4375	529	-3780	817	-3380	1942	-2325
22	-4370	538	-3775	829	-3375	1945	-2255
25	-4365	547	-3765	841	-3370	1948	-2210
46	-4360	553	-3760	844	-3360	1953	-2130
52	-4355	556	-3755	874	-3345		-1990
55	-4350	559	-3745	877	-3325		-1820

67	-4340	568	-3735	895	-3265		-1715
91	-4335	571	-3725	928	-3260		-710
118	-4330	586	-3720	970	-3250		-660
154	-4325	589	-3695	982	-3230		-580
202	-4320	601	-3690	991	-3210		1615

Figures (S1-S20)

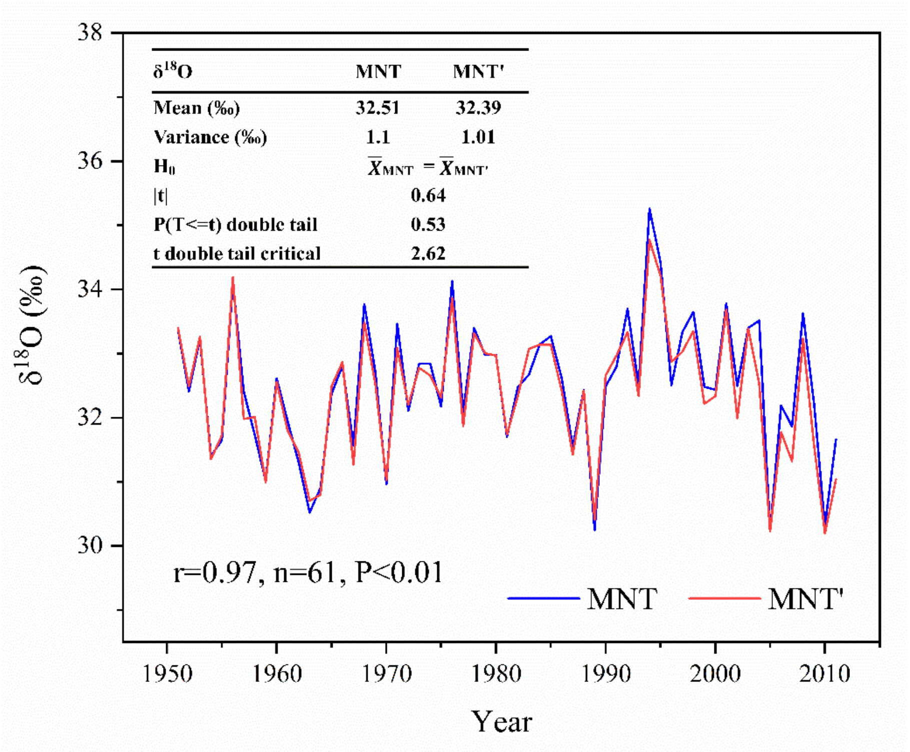

Fig. S1. Inter-laboratory comparison of $\delta^{18}O$ measurements from 10 trees analyzed in the laboratories in China (MNT, blue line) and Germany (MNT’, red line). The inserted table illustrates significance test results for the difference in $\delta^{18}O$ measurements between the laboratories at a level of α=0.01. No significant differences in $\delta^{18}O$ measurements were detected.

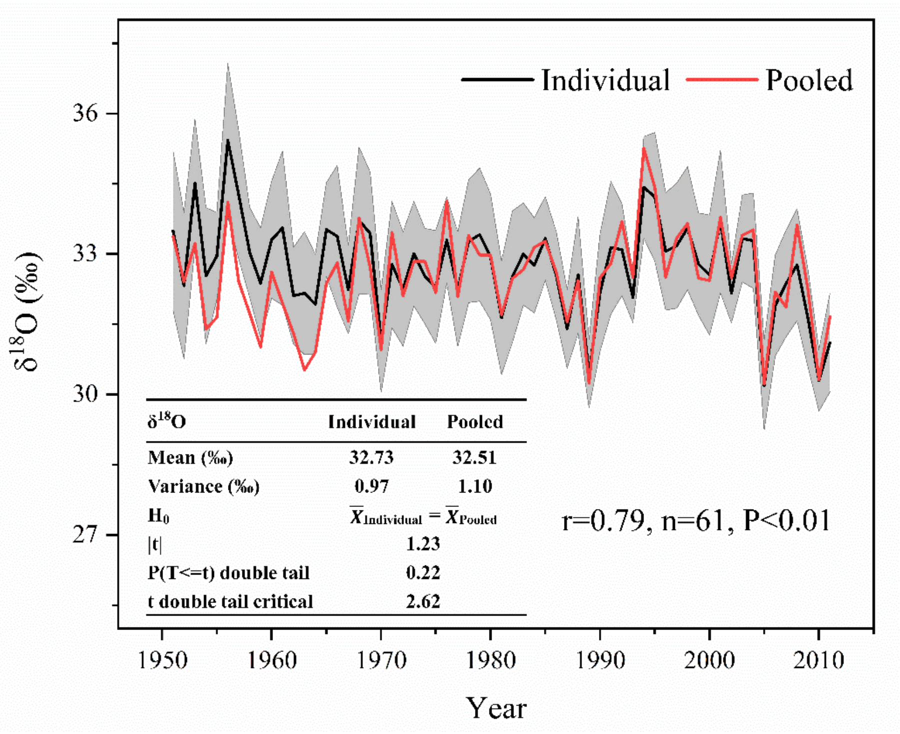

Fig. S2. Comparison of $\delta^{18}O$ measurements obtained via individual trees (black line) and pooling (red line) methods. The grey shaded area represents the 95% confidence intervals for individual series. The inset table shows the significance test results for the difference in $\delta^{18}O$ measured by the two methods, at a level of $\alpha=0.01$. There is no significant difference in $\delta^{18}O$ measurements.

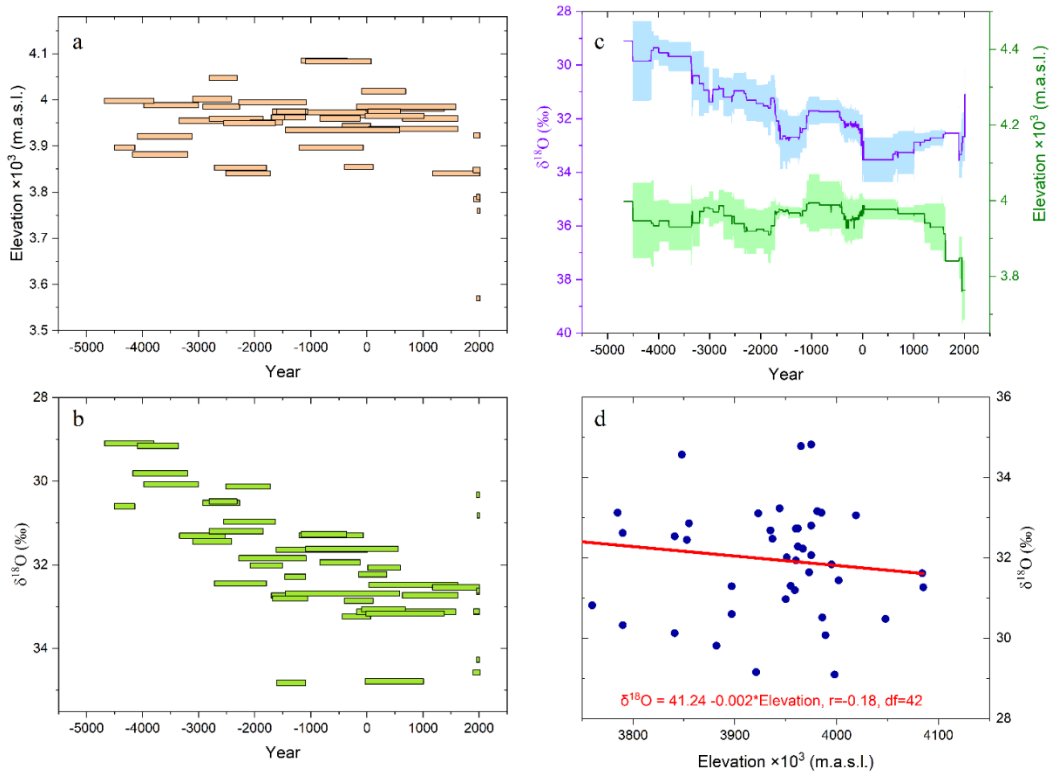

Fig. S3. Relationship between $\delta^{18}O$ series and sampling-tree elevation over the past 6700 years. (a) Sampling-tree elevations and time-series length of each individual tree. (b) Series mean $\delta^{18}O$ and time-series length of each individual tree. Each floating bar represents one $\delta^{18}O$ series and has a length indicating the length of the sample series. (c) Illustration of arithmetic means of the series in part (a) (green line) and part (b) (purple line) and the 95% confidence intervals (shading), respectively. (d) Scatterplot showing the overall relationship between the mean $\delta^{18}O$ values of each series and sampling-tree elevation. The relationship is not statistically significant.

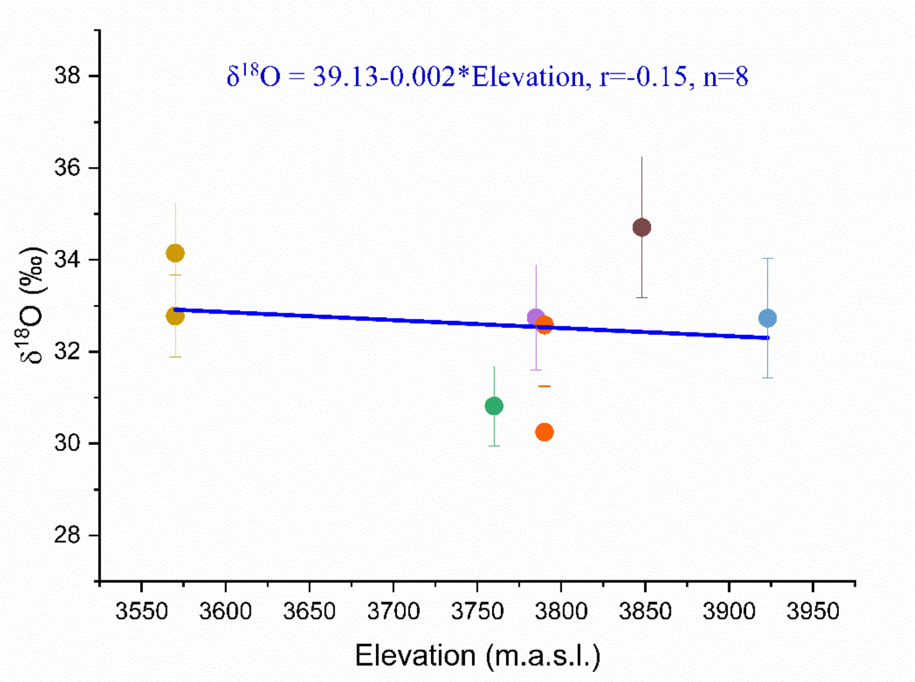

Fig. S4. Scatter plot illustrating the means (with ±1*SD) of eight $\delta^{18}O$ series against their respective sampling-tree elevations during 1964-2011 CE. These were the only eight $\delta^{18}O$ series for the interval 1964-2011 CE, excluding the pooled series. The analysis explores the potential effects of sampling elevation on the tree-ring $\delta^{18}O$, given uniform macroclimate conditions. The relationship is not statistically significant.

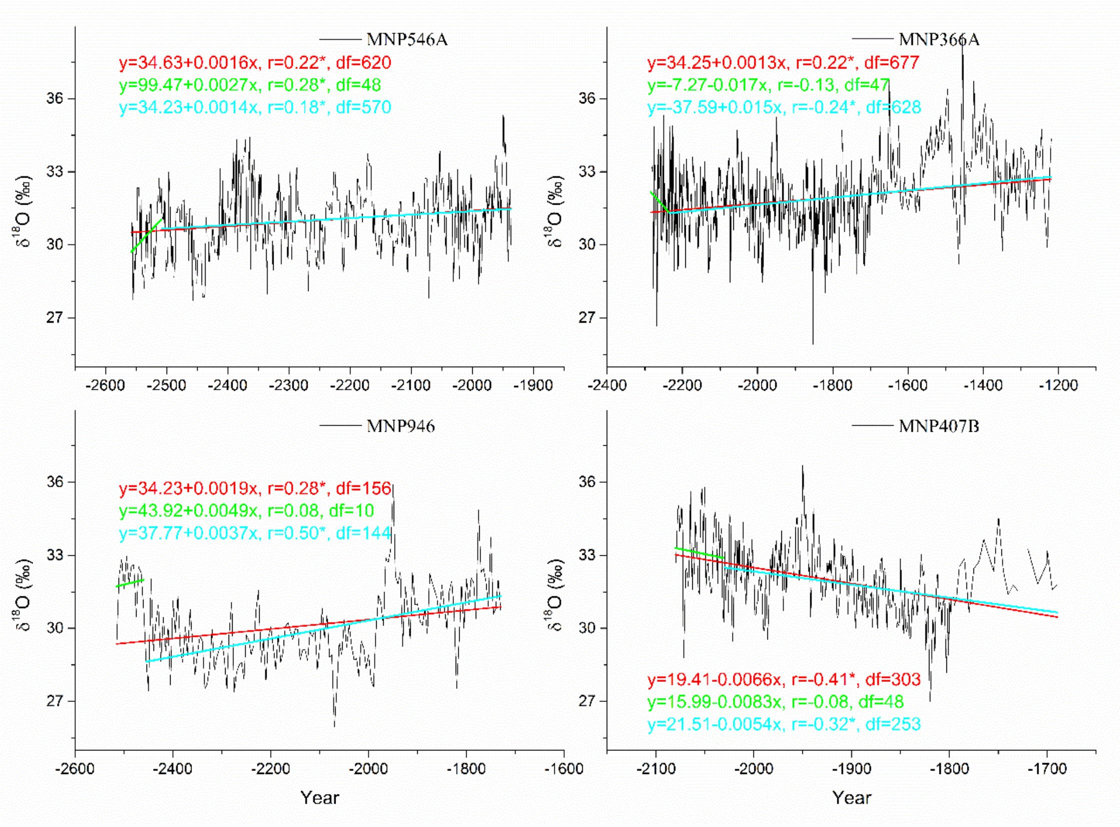
MNP546A
y=34.63+0.0016x, r=0.22*, df=620
y=99.47+0.0027x, r=0.28*, df=48
y=34.23+0.0014x, r=0.18*, df=570
MNP366A
y=34.25+0.0013x, r=0.22*, df=677
y=-7.27-0.017x, r=-0.13, df=47
y=-37.59+0.015x, r=-0.24*, df=628
MNP946
y=34.23+0.0019x, r=0.28*, df=156
y=43.92+0.0049x, r=0.08, df=10
y=37.77+0.0037x, r=0.50*, df=144
MNP407B
y=19.41-0.0066x, r=-0.41*, df=303
y=15.99-0.0083x, r=-0.08, df=48
y=21.51-0.0054x, r=-0.32*, df=253
δ18O (‰)
Year

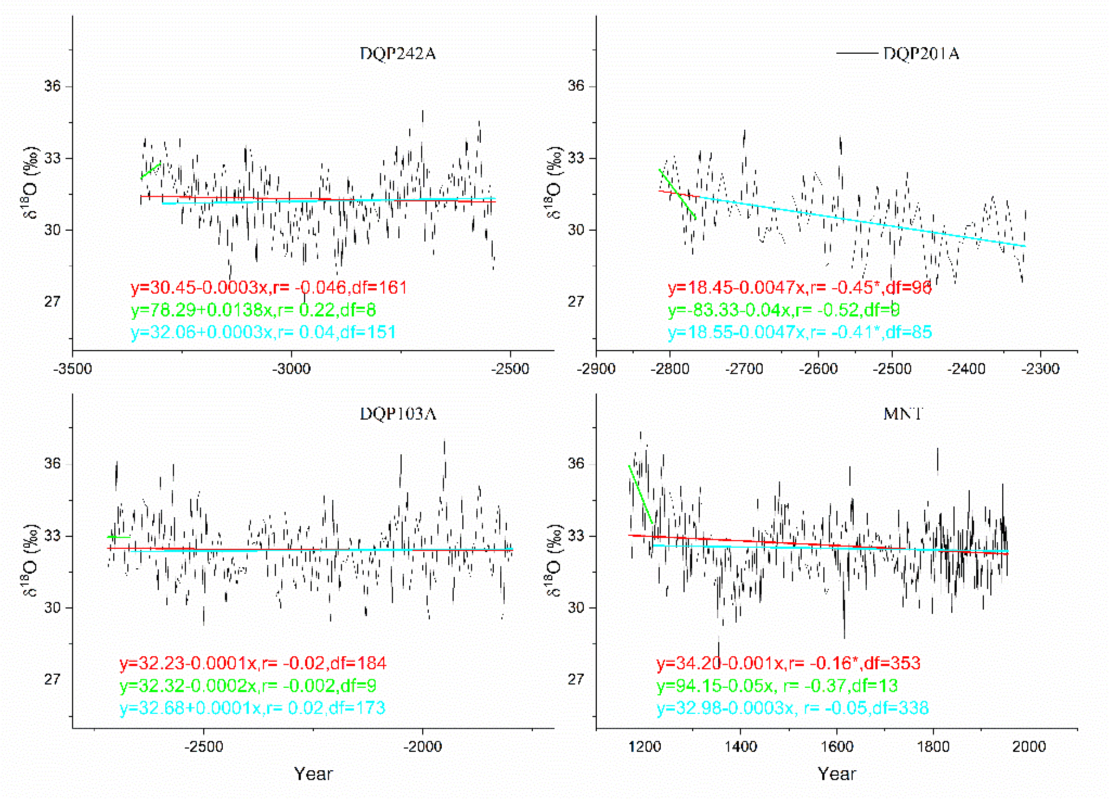
DQP242A
y=30.45-0.0003x,r= -0.046,df=161
y=78.29+0.0138x,r= 0.22,df=8
y=32.06+0.0003x,r= 0.04,df=151
DQP201A
y=18.45-0.0047x,r= -0.45*,df=96
y=-83.33-0.04x,r= -0.52,df=8
y=18.55-0.0047x,r= -0.41*,df=85
DQP103A
y=32.23-0.0001x,r= -0.02,df=184
y=32.32-0.0002x,r= -0.002,df=9
y=32.68+0.0001x,r= 0.02,df=173
MNT
y=34.20-0.001x,r= -0.16*,df=353
y=94.15-0.05x, r= -0.37,df=13
y=32.98-0.0003x, r= -0.05,df=338
δ18O (‰)
Year

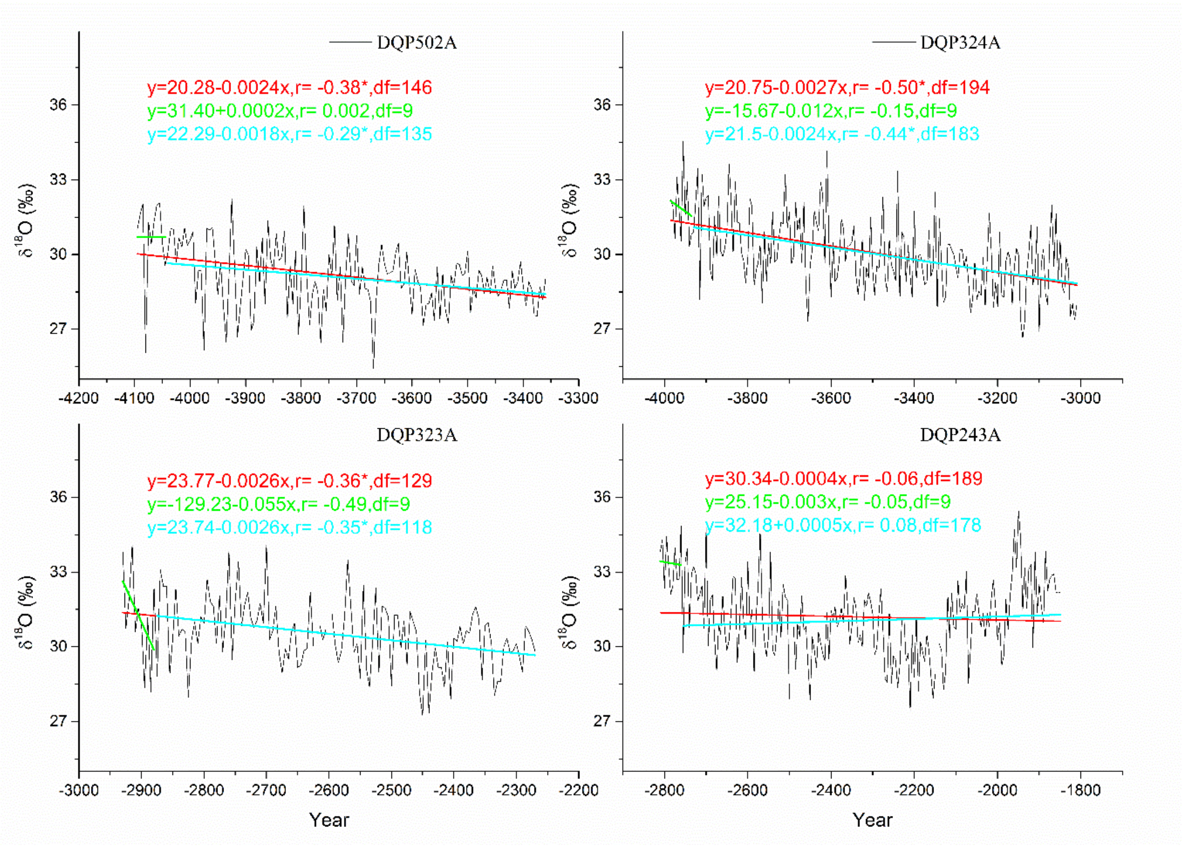
DQP502A
y=20.28-0.0024x,r= -0.38*,df=146
y=31.40+0.0002x,r= 0.002,df=9
y=22.29-0.0018x,r= -0.29*,df=135
DQP324A
y=20.75-0.0027x,r= -0.50*,df=194
y=-15.67-0.012x,r= -0.15,df=9
y=21.5-0.0024x,r= -0.44*,df=183
DQP323A
y=23.77-0.0026x,r= -0.36*,df=129
y=-129.23-0.055x,r= -0.49,df=9
y=23.74-0.0026x,r= -0.35*,df=118
DQP243A
y=30.34-0.0004x,r= -0.06,df=189
y=25.15-0.003x,r= -0.05,df=9
y=32.18+0.0005x,r= 0.08,df=178
δ18O (‰)
Year

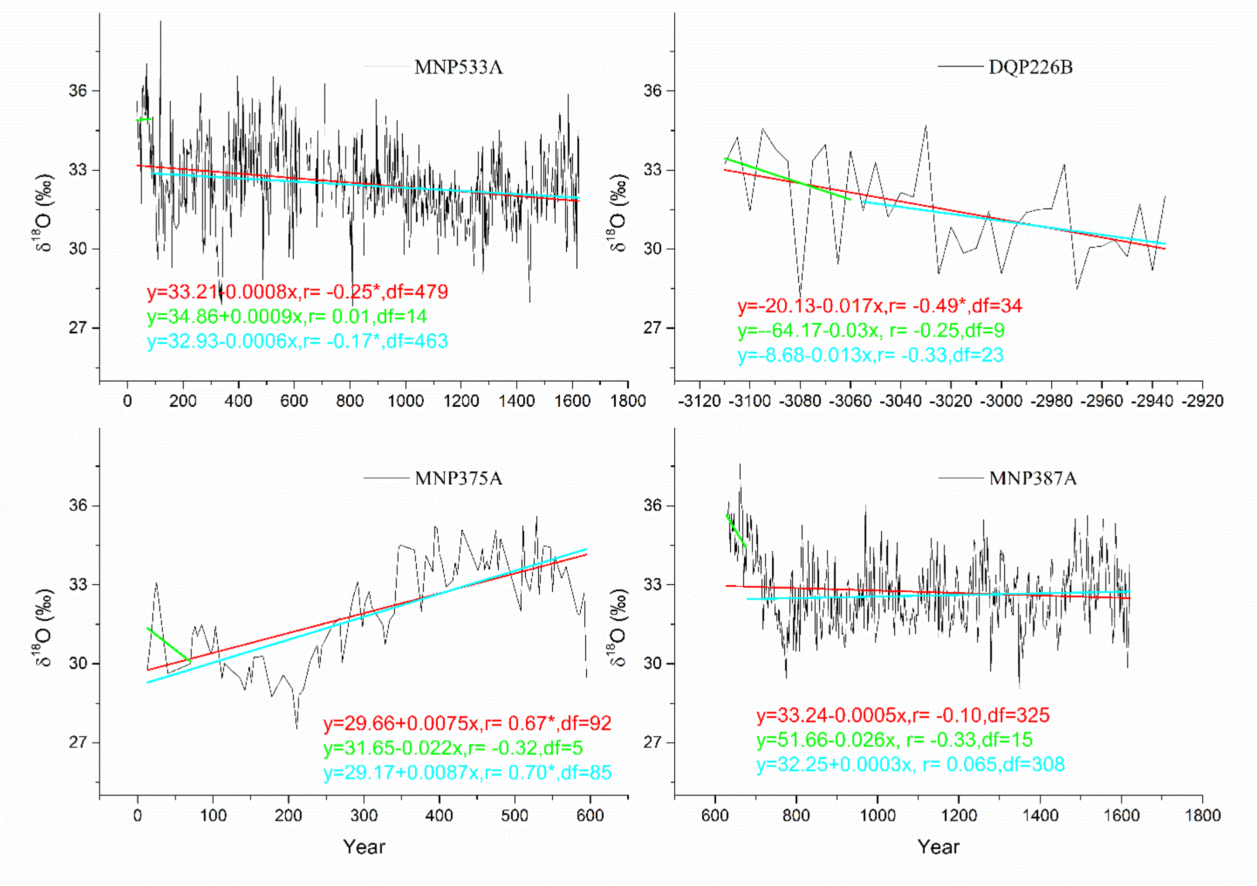
MNP533A
y=33.21-0.0008x,r= -0.25*,df=479
y=34.86+0.0009x,r= 0.01,df=14
y=32.93-0.0006x,r= -0.17*,df=463
DQP226B
y=-20.13-0.017x,r= -0.49*,df=34
y=--64.17-0.03x, r= -0.25,df=9
y=-8.68-0.013x,r= -0.33,df=23
MNP375A
y=29.66+0.0075x,r= 0.67*,df=92
y=31.65-0.022x,r= -0.32,df=5
y=29.17+0.0087x,r= 0.70*,df=85
MNP387A
y=33.24-0.0005x,r= -0.10,df=325
y=51.66-0.026x, r= -0.33,df=15
y=32.25+0.0003x, r= 0.065,df=308
δ18O (‰)
Year

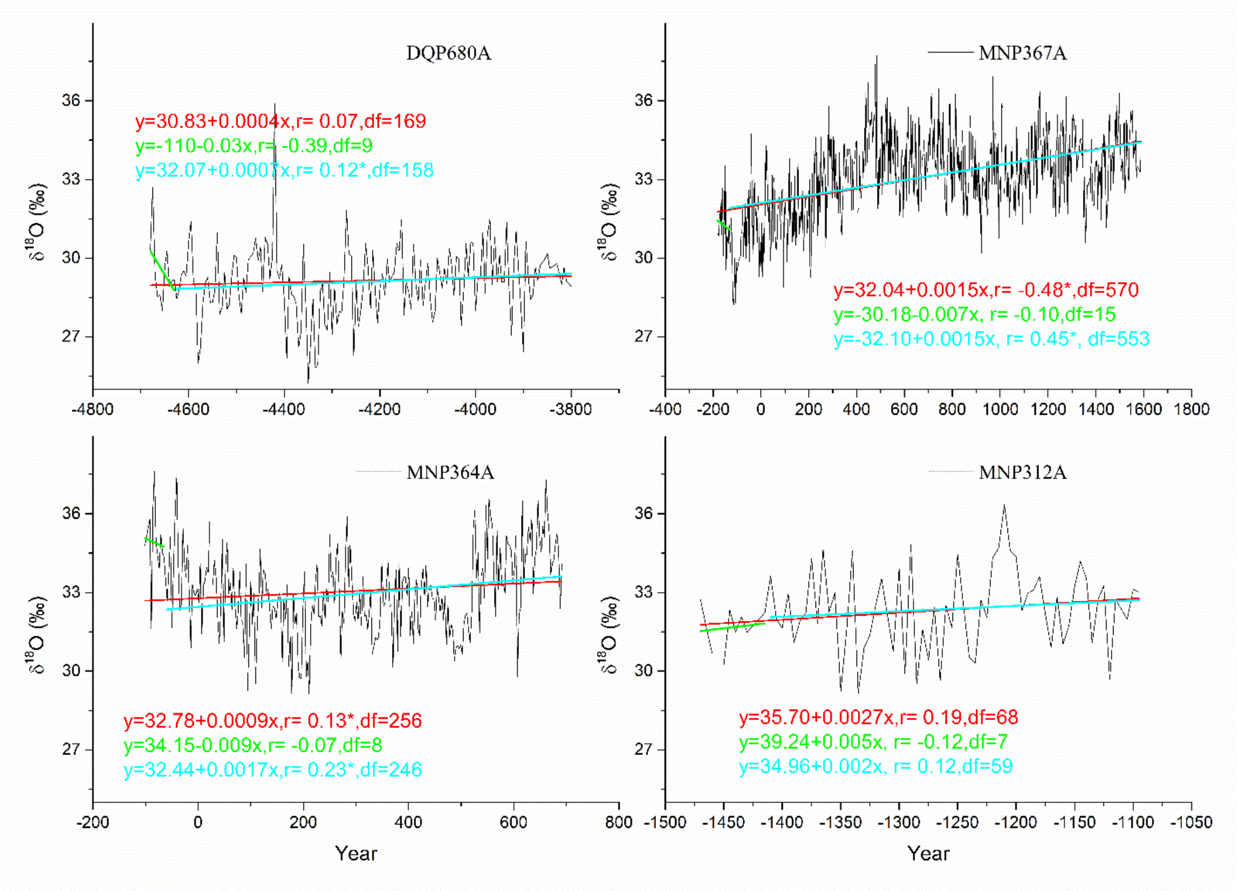
DQP680A
y=30.83+0.0004x,r= 0.07,df=169
y=-110-0.03x,r= -0.39,df=9
y=32.07+0.0007x,r= 0.12*,df=158
MNP367A
y=32.04+0.0015x,r= -0.48*,df=570
y=-30.18-0.007x, r= -0.10,df=15
y=-32.10+0.0015x, r= 0.45*, df=553
MNP364A
y=32.78+0.0009x,r= 0.13*,df=256
y=34.15-0.009x,r= -0.07,df=8
y=32.44+0.0017x,r= 0.23*,df=246
MNP312A
y=35.70+0.0027x,r= 0.19,df=68
y=39.24+0.005x, r= -0.12,df=7
y=34.96+0.002x, r= 0.12,df=59
δ18O (‰)
Year

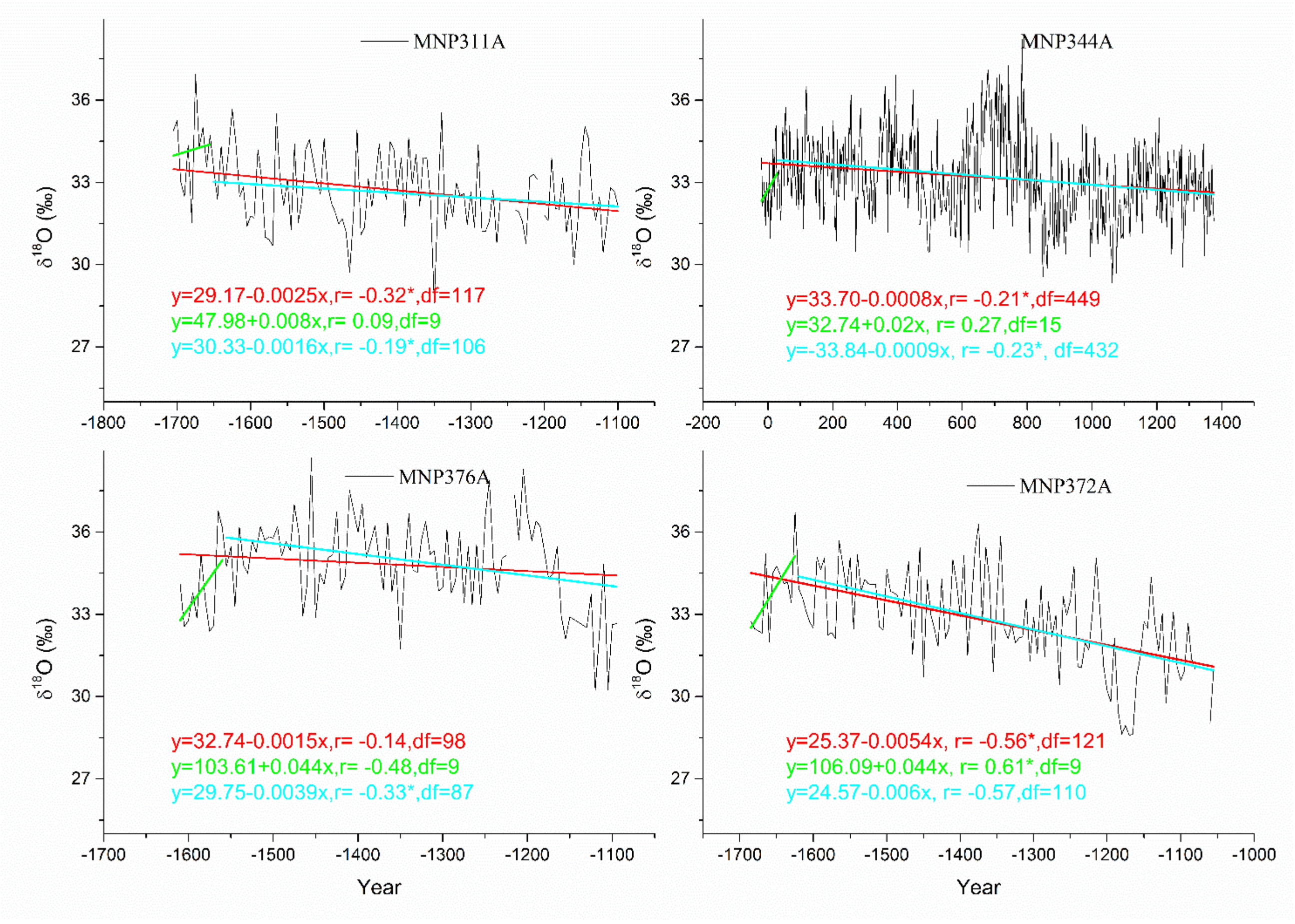
MNP311A
y=29.17-0.0025x,r= -0.32*,df=117
y=47.98+0.008x,r= 0.09,df=9
y=30.33-0.0016x,r= -0.19*,df=106
MNP344A
y=33.70-0.0008x,r= -0.21*,df=449
y=32.74+0.02x, r= 0.27,df=15
y=-33.84-0.0009x, r= -0.23*, df=432
MNP376A
y=32.74-0.0015x,r= -0.14,df=98
y=103.61+0.044x,r= -0.48,df=9
y=29.75-0.0039x,r= -0.33*,df=87
MNP372A
y=25.37-0.0054x, r= -0.56*,df=121
y=106.09+0.044x, r= 0.61*,df=9
y=24.57-0.006x, r= -0.57,df=110
δ18O (‰)
Year

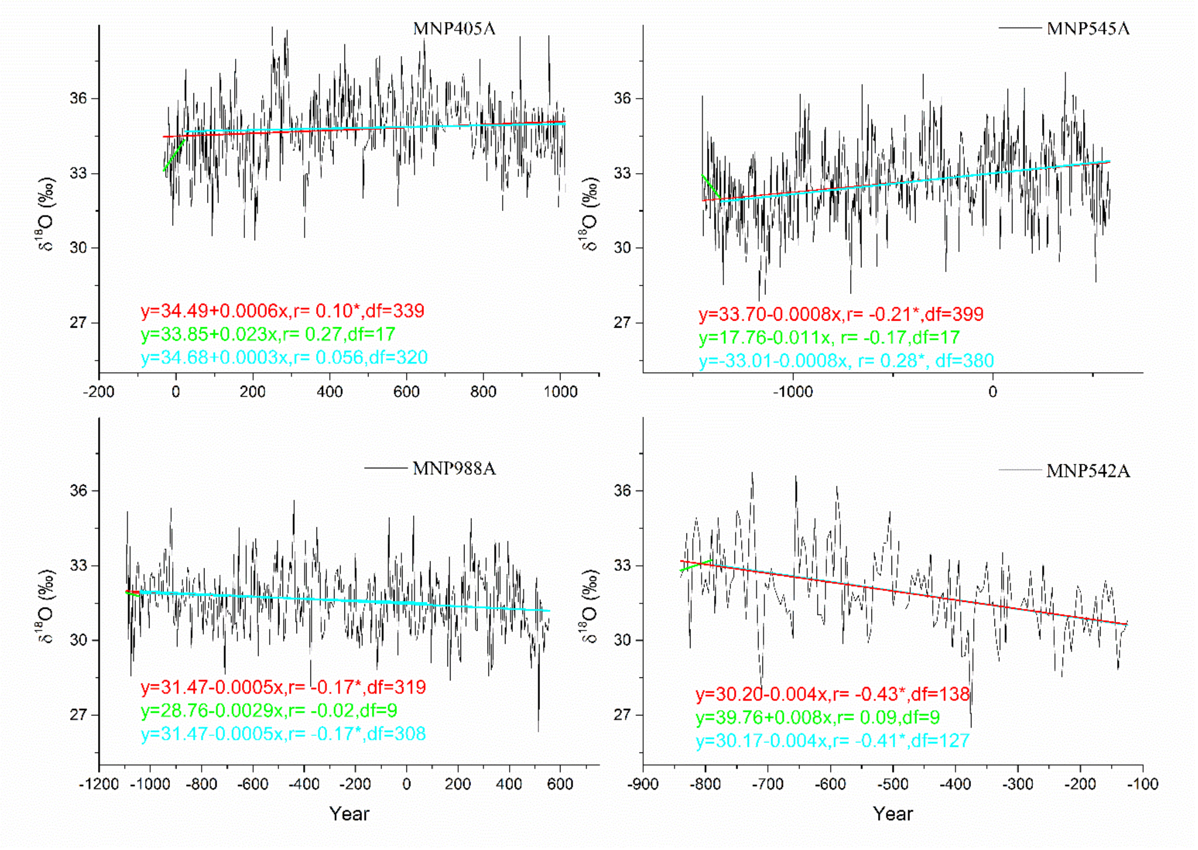
MNP405A
y=34.49+0.0006x,r= 0.10*,df=339
y=33.85+0.023x,r= 0.27,df=17
y=34.68+0.0003x,r= 0.056,df=320
MNP545A
y=33.70-0.0008x,r= -0.21*,df=399
y=17.76-0.011x, r= -0.17,df=17
y=-33.01-0.0008x, r= 0.28*, df=380
MNP988A
y=31.47-0.0005x,r= -0.17*,df=319
y=28.76-0.0029x,r= -0.02,df=9
y=31.47-0.0005x,r= -0.17*,df=308
MNP542A
y=30.20-0.004x,r= -0.43*,df=138
y=39.76+0.008x,r= 0.09,df=9
y=30.17-0.004x,r= -0.41*,df=127
δ18O (‰)
Year

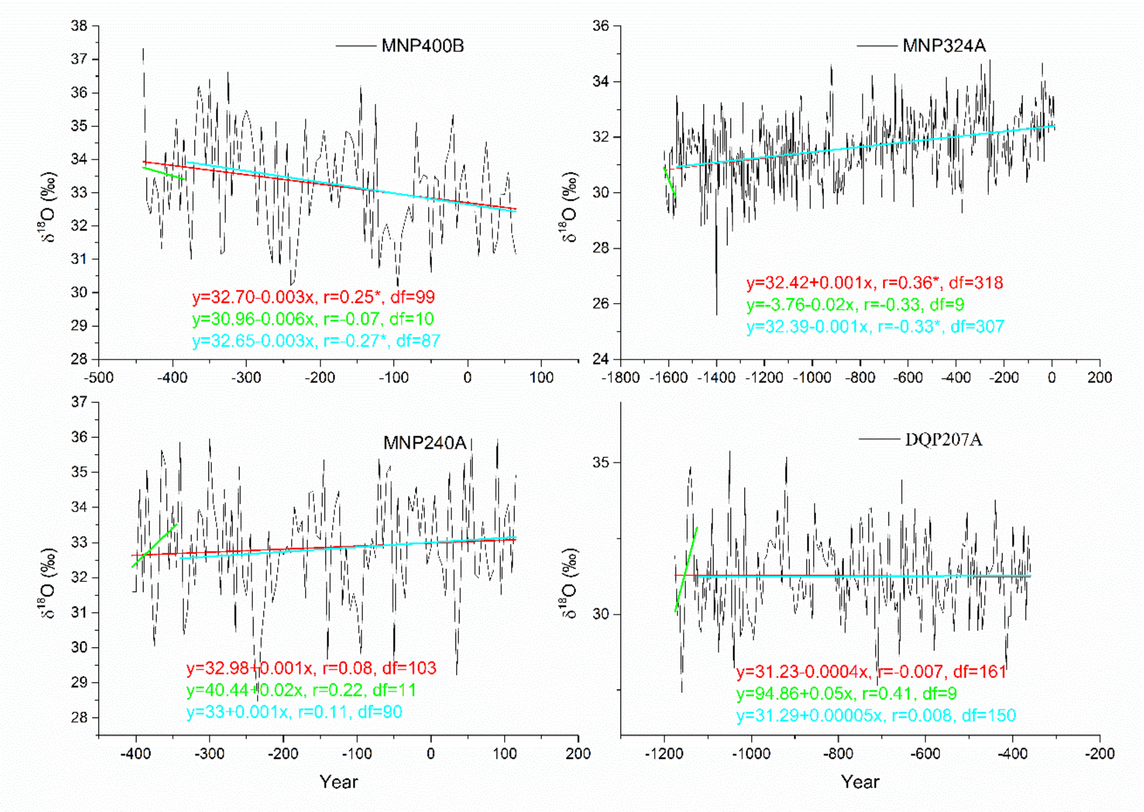
MNP400B
y=32.70-0.003x, r=0.25*, df=99
y=30.96-0.006x, r=-0.07, df=10
y=32.65-0.003x, r=-0.27*, df=87
MNP324A
y=32.42+0.001x, r=0.36*, df=318
y=-3.76-0.02x, r=-0.33, df=9
y=32.39-0.001x, r=-0.33*, df=307
MNP240A
y=32.98+0.001x, r=0.08, df=103
y=40.44+0.02x, r=0.22, df=11
y=33+0.001x, r=0.11, df=90
DQP207A
y=31.23-0.0004x, r=-0.007, df=161
y=94.86+0.05x, r=0.41, df=9
y=31.29+0.00005x, r=0.008, df=150
δ18O (‰)
Year

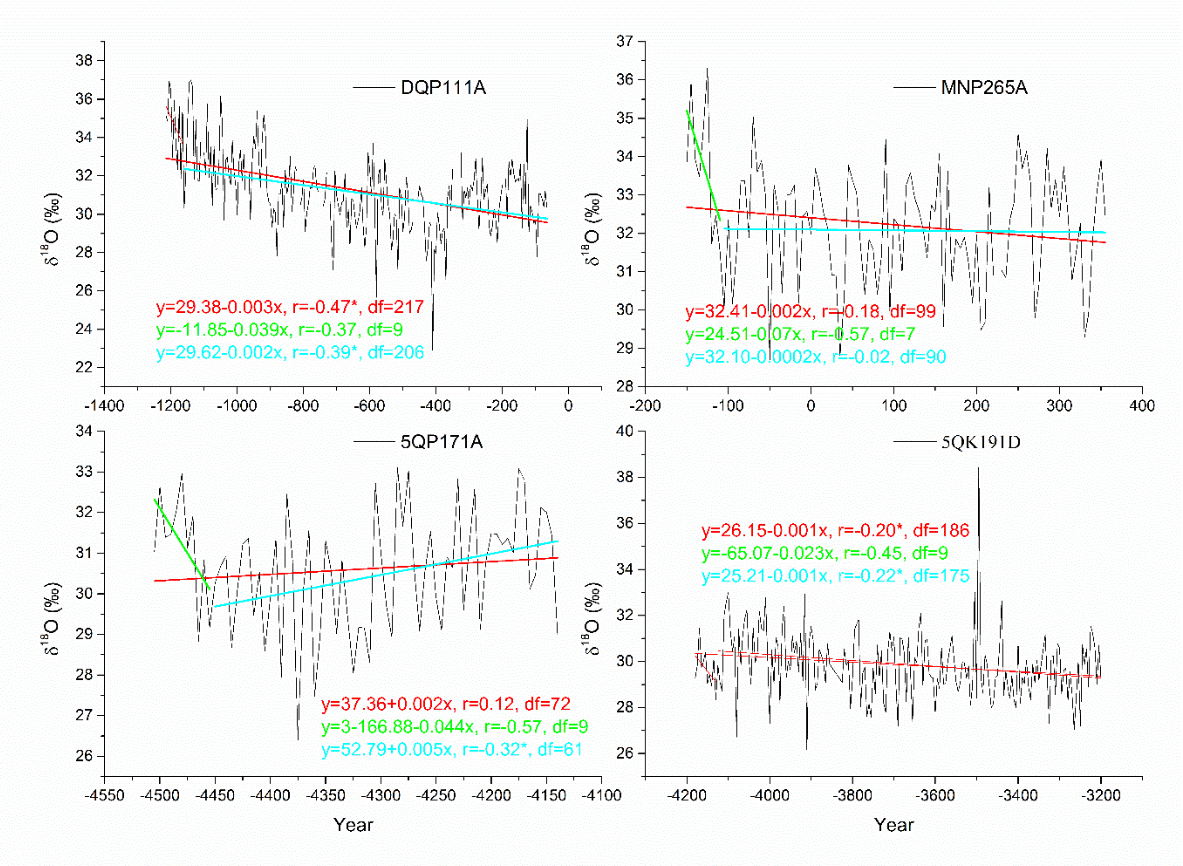

Fig. S5. Quantification of potential juvenile age effects in all 36 $\delta^{18}O$ series (excluding tree samples that did not have pith offset estimates), via linear fitting of each single $\delta^{18}O$ series in three segments. Scale 1 (red) uses the whole series; Scale 2 (green) uses the first 50 years of tree growth; Scale 3 (cyan) uses the remaining part (older than 50 years). The regression formulae, the Pearson correlation coefficient (r), and degrees of freedom (df) for the linear fit between $\delta^{18}O$ and calendar year are presented in corresponding colors. The star (*) indicates that r was significant at the 0.05 level.

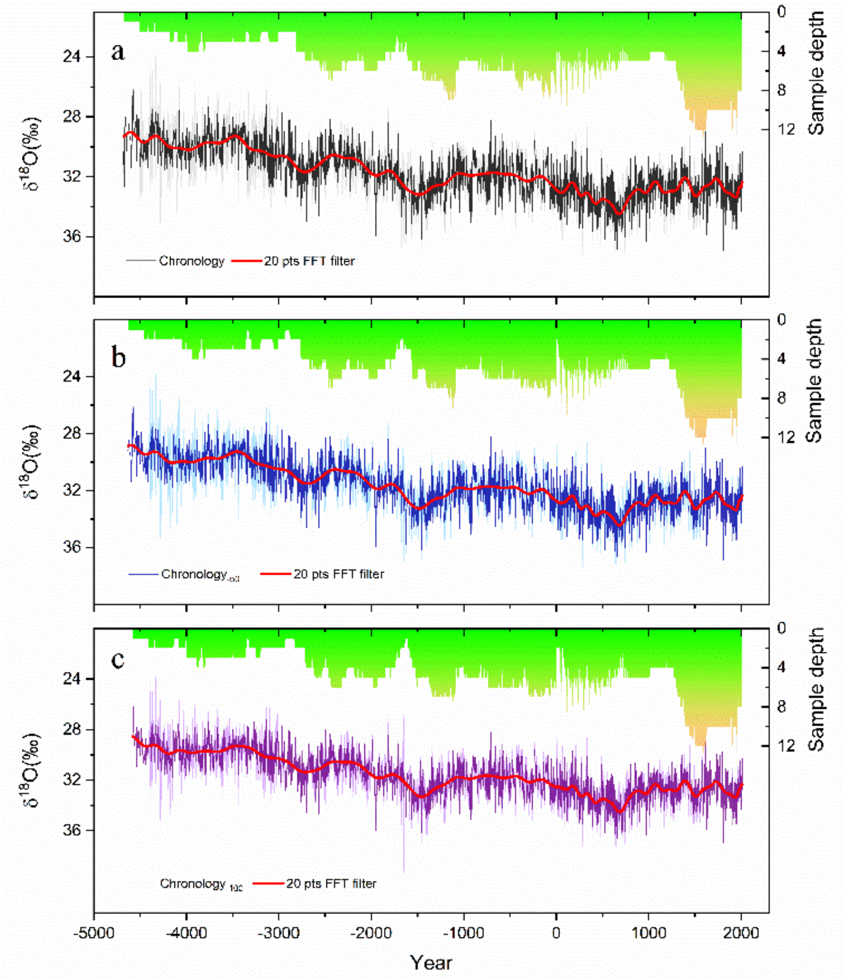

Fig. S6. Sensitivity analysis for the existence of potential juvenile age effects in the δ18O chronology. (a) The thin black line is the original chronology, the arithmetic mean of all δ18O series. (b) Chronology-50 (thin blue line) is the arithmetic mean of all series after removing the first 50 years of each series (except those in the most recent 100 years). (c) Chronology-100 (thin purple line) is the arithmetic mean of all series after removing the first 100 years of each series (except those in the most recent 100 years). The biological age of the series in the most recent 100 years was older than 300 years. The pooled series MNT was removed from the first 50 years of the longest series (MNT710B), but there was no possibility to remove the others. The bold red lines are their corresponding curves smoothed using a 20-point FFT (Fast Fourier Transform). Shading indicates the 95% confidence intervals of the unsmoothed series. Sample size for each series is shown at the top of each panel (right-hand scales).

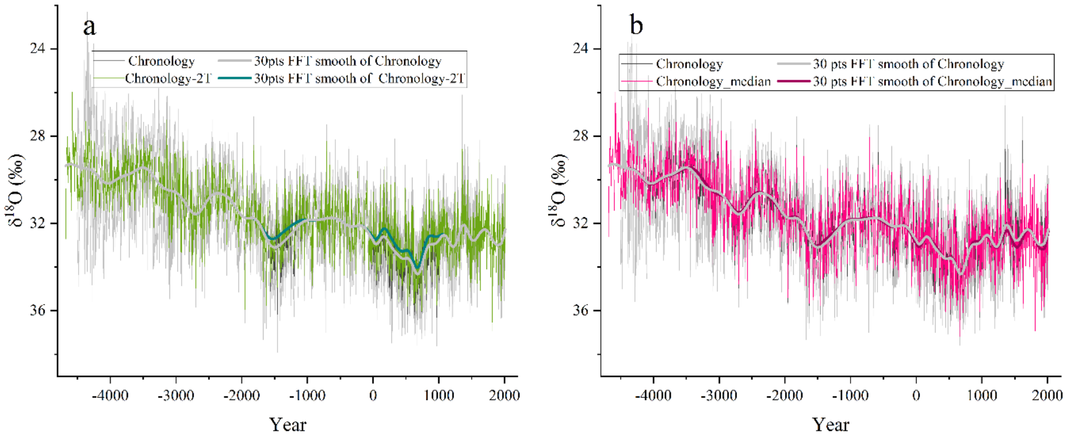

Fig. S7. Impact of level offsets between individual tree-ring $\delta^{18}O$ time series on the mean isotope chronology. (a) Comparison between the original chronology (the arithmetic mean of all $\delta^{18}O$ series, dark gray line) and the test chronology (named "chronology-2T", light green line) in case of removing the samples MNP376A and MNP405A (treated as outliers). (b) Comparison between the original mean chronology (thin black line) and the median chronology (named as "chronology_median", pink line), for which we calculated the median value (rather than mean) of all individual chronologies in order to reduce the effect of outliers. The thick lines are their corresponding low-pass filter series, smoothed using a 30-point Fast Fourier Transform (FFT). Shading indicates the 95% confidence intervals of the unsmoothed series.

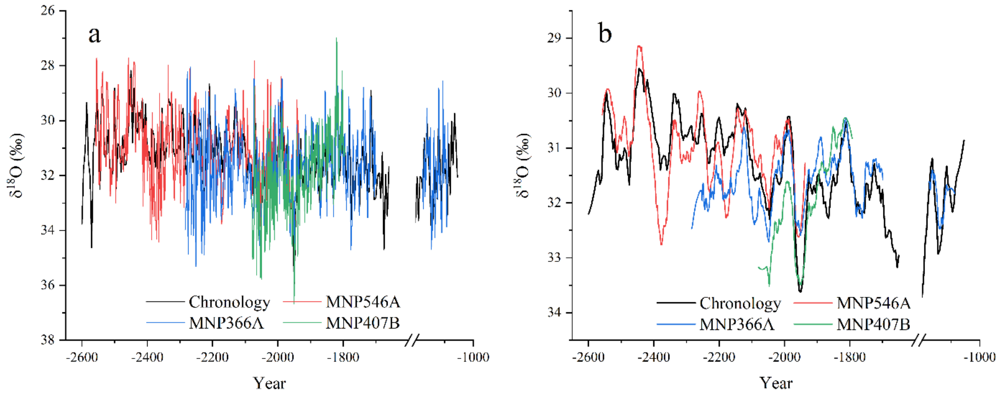

Fig. S8. Comparison of the tree-ring DLH δ^{18}O chronology (5-year resolution) with time series (annual resolution) of three individual δ^{18}O records during 2550 BCE–1100 CE (a) and their corresponding 50-year low-pass filter curves (b). The individual annual δ^{18}O records generally track the direction of the DLH mean δ^{18}O chronology with 5-year resolution. This agreement suggests that the composite chronology is insensitive to individual δ^{18}O records with different time resolutions.

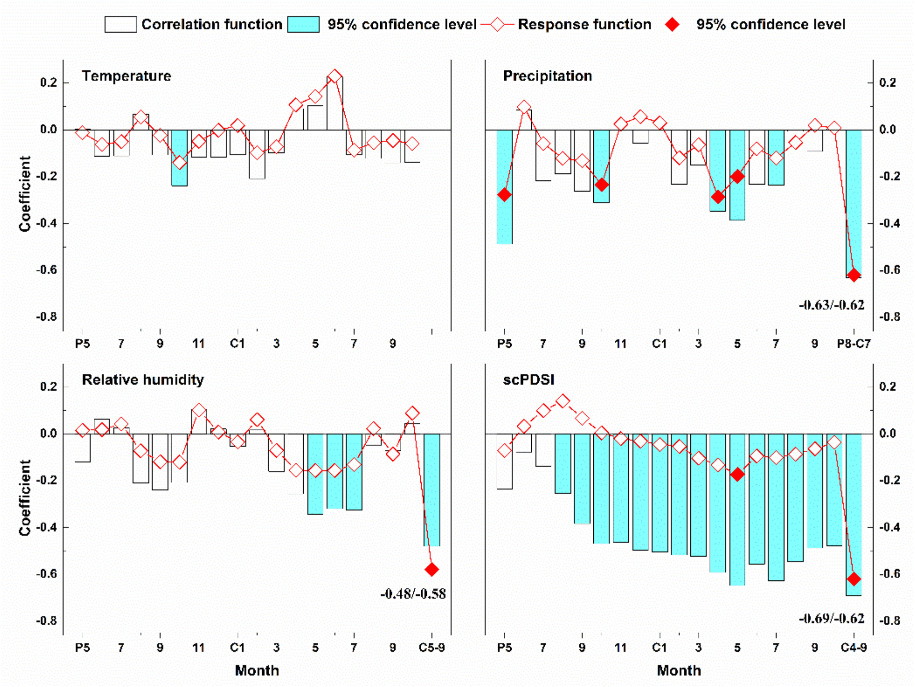

Fig. S9. Pearson correlation coefficients and response functions between the mean DLH δ^{18}O chronology and monthly mean temperature, precipitation, RH, scPDSI, and optimal seasonal combination (with the strongest correlation) for the period 1956–2011 CE. The numbers annotated on the precipitation, RH, and scPDSI panels are the Pearson correlation coefficients with the optimal seasonal mean using the raw data and using first-order differences respectively. Significant values at the 95% confidence level are marked by colored symbols.

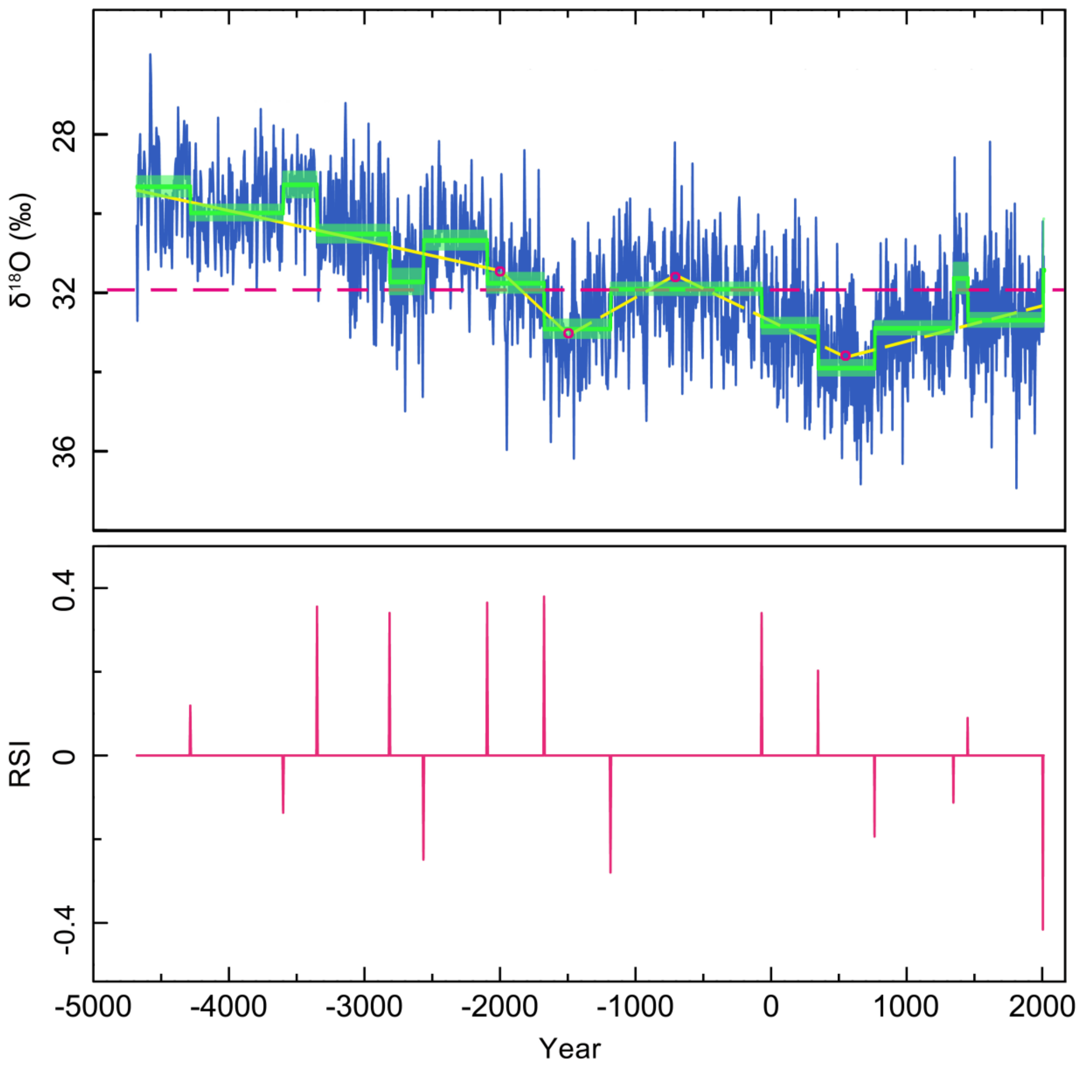

Fig. S10. **Results of trend-point analysis and the regime shift analysis (STARS) to detect shifts in the trend and mean for the DLH $\delta^{18}O$ chronology, as explained in the Supplementary Methods.** Upper panel shows the time series (blue) and regimes identified either by piecewise continuous linear trends (yellow lines) and their change points (magenta circles) or by shifts in the mean detected by STARS (green lines). Significant changes in temporal trends of the time series were identified using the "segmented" package in the R environment (25, 26) with change point years of $p < 0.05$ (544 CE, 709 BCE, 1501 BCE, 2000 BCE) (magenta circles). The mean value of the time series is shown by the red dashed line. Shift points for the mean were identified by STARS with $p \leqslant 0.01$ and are shown in the lower panel as the Regime Shift Index (RSI) and listed in Table S7.

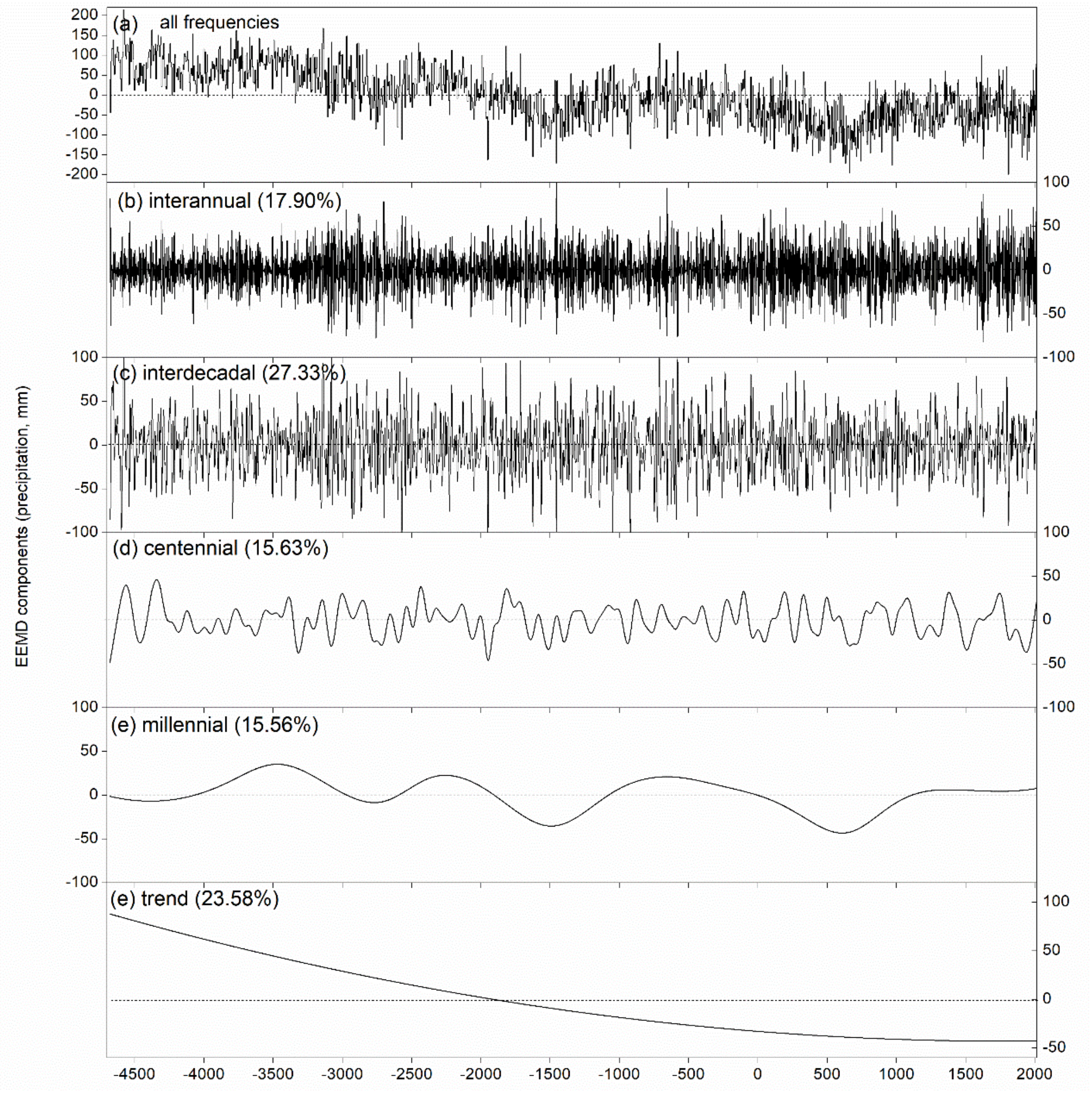

Fig. S11. Intrinsic temporal components of our DHL tree-ring δ^{18}O precipitation reconstruction by ensemble empirical mode decomposition analysis (EEMD). The raw data was interpolated annually by using a linear interpolation method before the EEMD analysis. We defined the inter-annual component (1–10 years) as sum of variability, interdecadal component (10–100 years), centennial component (100–1000 years), millennial component (> 1000 years) for variability and the secular trend as the last component. The 1024-year cycle (8.1%) dominates the millennial-scale spectral range. A ratio of the standard deviation of the added noise and that of data is set to 0.2 and an ensemble number is set to 100 for ensemble empirical mode decomposition calculation.

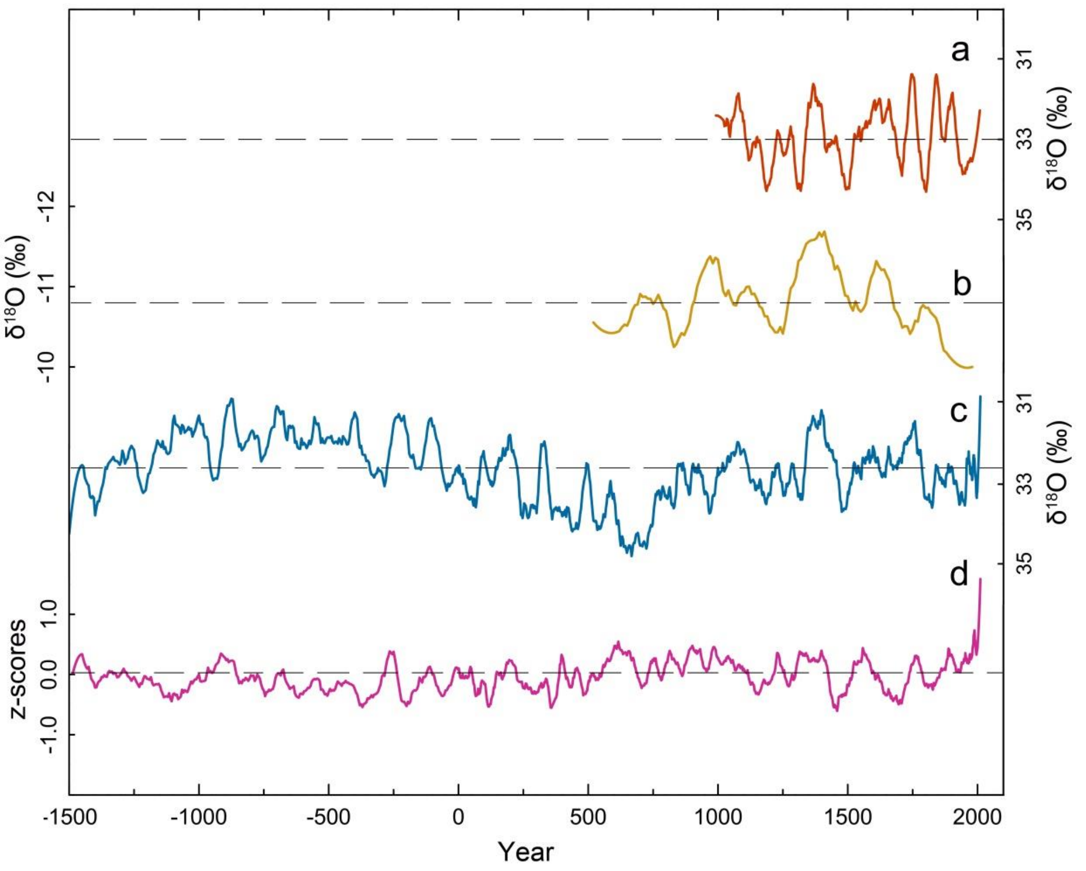

Fig. S12. (a) Tree-ring $\delta^{18}O$ raw series from the forest stand (37°16′N, 97°32′E, 4000 m a.s.l.) in Delingha, located approximately 50 km from our study site (8). (b) $\delta^{18}O$ record from the Dunde ice core (26). (c) Mean tree-ring $\delta^{18}O$ raw series established in this study (DLH $\delta^{18}O$). (d) Signal-free regional curve standardization of a tree-ring width chronology from the northeastern TP (1). The black dashed lines represent the mean of each time series (with respect to common time period of 990-1980 CE). Time series in a, c and d were interpolated to a resolution consistent with the Dunde oxygen isotope sequence (10 years); correlation analysis for the common time period (990-1980 CE) then showed pairs a and c, b and c, and c and d were all significantly correlated (A and C, 0.51, p <0.01; B and C, 0.22, p <0.05; C and D, -0.39, p <0.01).

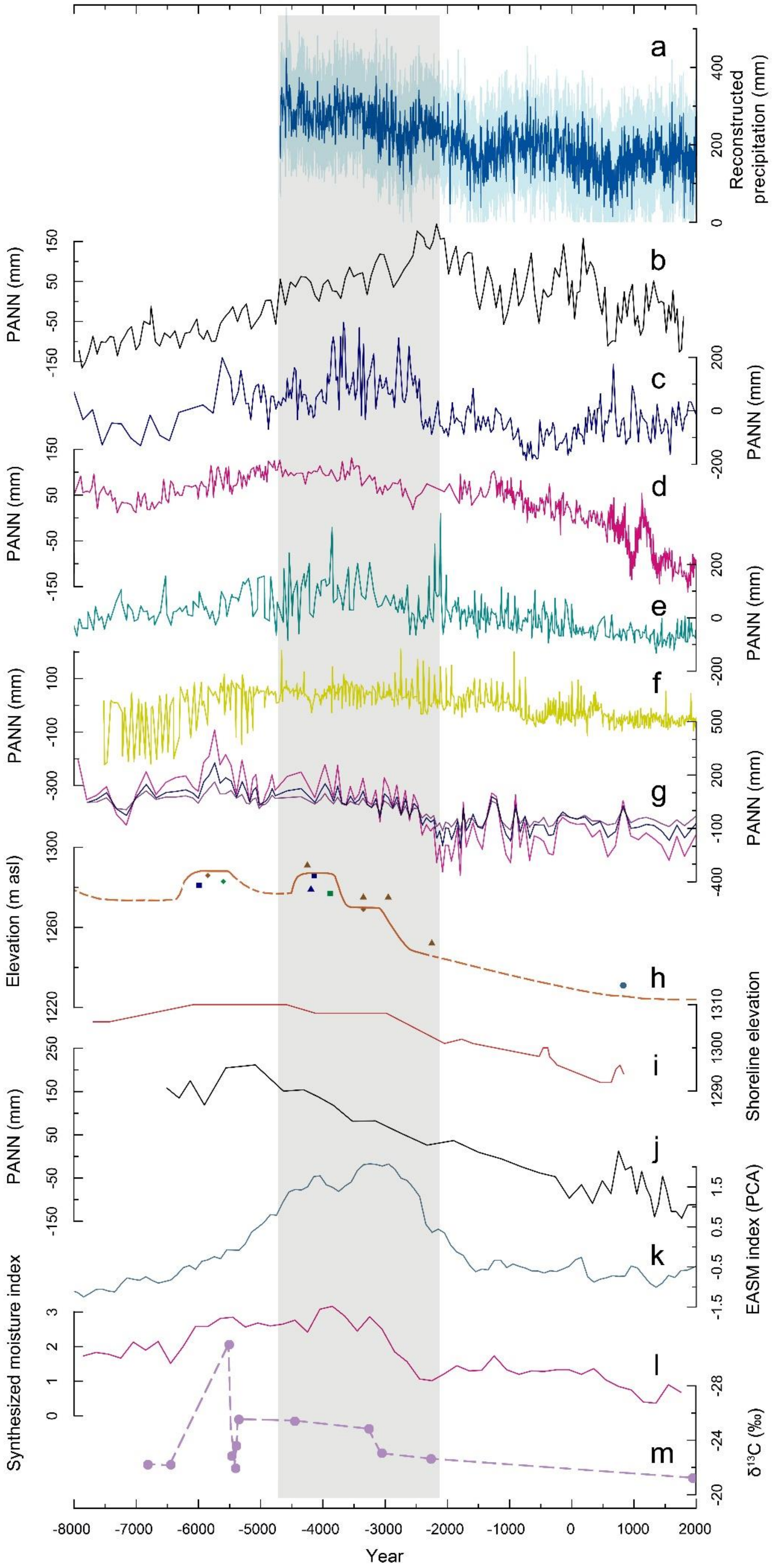

a
Reconstructed precipitation (mm)
0
200
400
b
PANN (mm)
-150
-50
50
150
c
PANN (mm)
-200
0
200
d
PANN (mm)
-150
-50
50
150
e
PANN (mm)
-200
0
200
f
PANN (mm)
-300
-100
100
g
PANN (mm)
-400
-100
200
500
h
Elevation (m asl)
1220
1260
1300
i
Shoreline elevation
1290
1300
1310
j
PANN (mm)
-150
-50
50
150
250
k
EASM index (PCA)
-1.5
-0.5
0.5
1.5
l
Synthesized moisture index
0
1
2
3
m
δ13C (‰)
-20
-24
-28
-8000
-7000
-6000
-5000
-4000
-3000
-2000
-1000
0
1000
2000
Year

Fig. S13. Comparison of the DLH $\delta^{18}O$ precipitation reconstruction (a, this study) with other regional ASM hydroclimate reconstructions. b, pollen-based mean annual precipitation deviations relative to 8000 BCE–1800 CE from Sihailongwan Maar Lake (29); c, pollen-based annual precipitation deviations relative to Holocene mean from Daihai Lake (note their method uses weighted averaging and partial least squares) (30); d, pollen-based Gonghai Lake annual precipitation deviations from the Holocene mean (31); e, pollen-based mean annual precipitation deviations from the Holocene mean for lakes Dalianhai and Qinghai on the Northeastern Qinghai-Tibetan Plateau (32); f, pollen-based East Asian summer monsoon precipitation for northern China and KCM-simulated East Asian summer monsoon precipitation deviations from the Holocene mean (33); g, pollen-based annual precipitation (red), extended summer precipitation from May to September (dark blue) and summer precipitation from June to August (purple) from the Holocene mean in the ASM region (34); h, Lake Dali level for the past 10000 years, based on lake sediments (squares), alluvial deposits (triangles) and beach ridges (diamonds) dated using ^{14}C from shells (blue), ^{14}C from charcoal (green), and OSL (brown) (35); i, moisture history at the ASM margin derived from shorelines or lacustrine sequences of Baijian Lake (36); j, pollen-based mean annual precipitation deviations relative to 1970–6400 BCE from Xingyun Lake (37); k, ASM index synthesized from monsoonal eastern China (38); l, pollen-based moisture index synthesized for the ASM rainfall belt over northern China (39). m, the $\delta^{13}C$ record of buried wood fragments (*Picea crassifolia* Kom.) from the Qinghai Lake basin (27). The shaded area covers the period from 4700–2000 BCE.

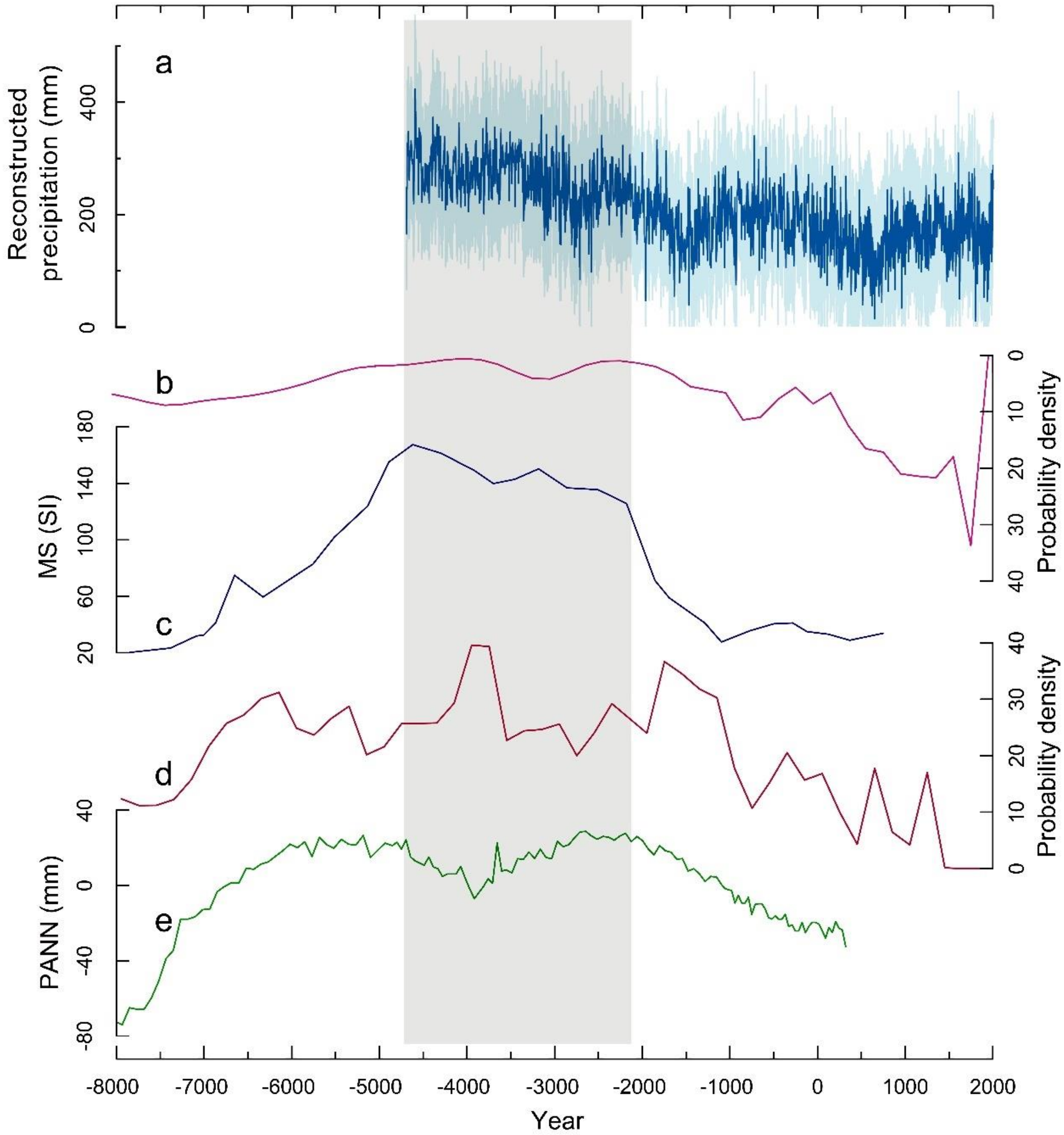

Fig. S14. (a) Tree-ring DLH $\delta^{18}O$ precipitation reconstruction (DLH $\delta^{18}O$; this study); (b) ASM evolution during the Holocene recorded by the frequency distribution of aeolian sand in China's four main sandy lands (Hulun Buir, Otindag, Horqin, Mu Us) and based on 88 dates from 44 sites (40); (c) ASM precipitation variations reconstructed using magnetic susceptibility (MS) records from the Yulin loess-paleosol section based on 3 dates (41); (d) ASM history during the Holocene reconstructed using the probability density of palaeosol dates from the Chinese Loess Plateau based on 348 dates (40); (e) reconstructed Holocene precipitation deviations relative to the mean value of 6000 BCE–300 CE from the Yaoxian loess-paleosol section, Guanzhong basin,

located on the southern Chinese Loess Plateau based on 7 dates (42). The shaded area covers the period from 4700–2000 BCE.

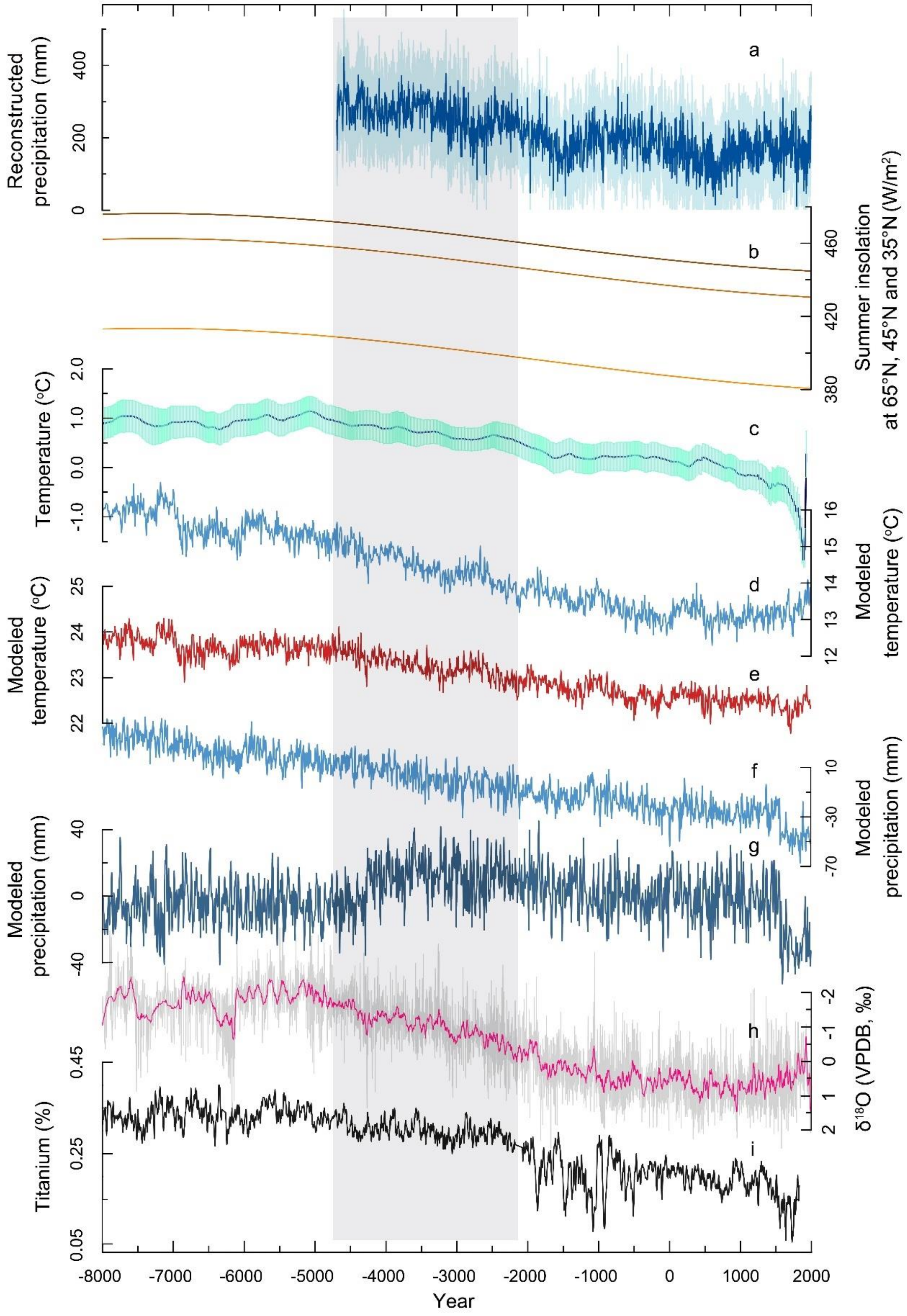

Fig. S15. (a) Tree-ring $\delta^{18}O$ precipitation reconstruction (DLH $\delta^{18}O$; this study); (b) summer insolation at 65°N,45°N and 35°N (43); (c) temperature anomalies for the extratropical Northern Hemisphere (44); (d) and (e), modeled mean annual temperature of northern China (30°-50°N, 90°-120°E) and southern China (20°-30°N, 90°-120°E)

(34); (f) and (g), modeled annual precipitation of northern China and southern China (17) from the Holocene mean; (h) normalized stalagmite composite $\delta^{18}O$ record from central China (see Supplementary Methods for more details) and the 30-y moving average (pink line); (i) Variation in location of the Intertropical Convergence Zone (ITCZ) reflected by Cariaco Basin Ti concentrations (45). The shaded area covers the period from 4700–2000 BCE.

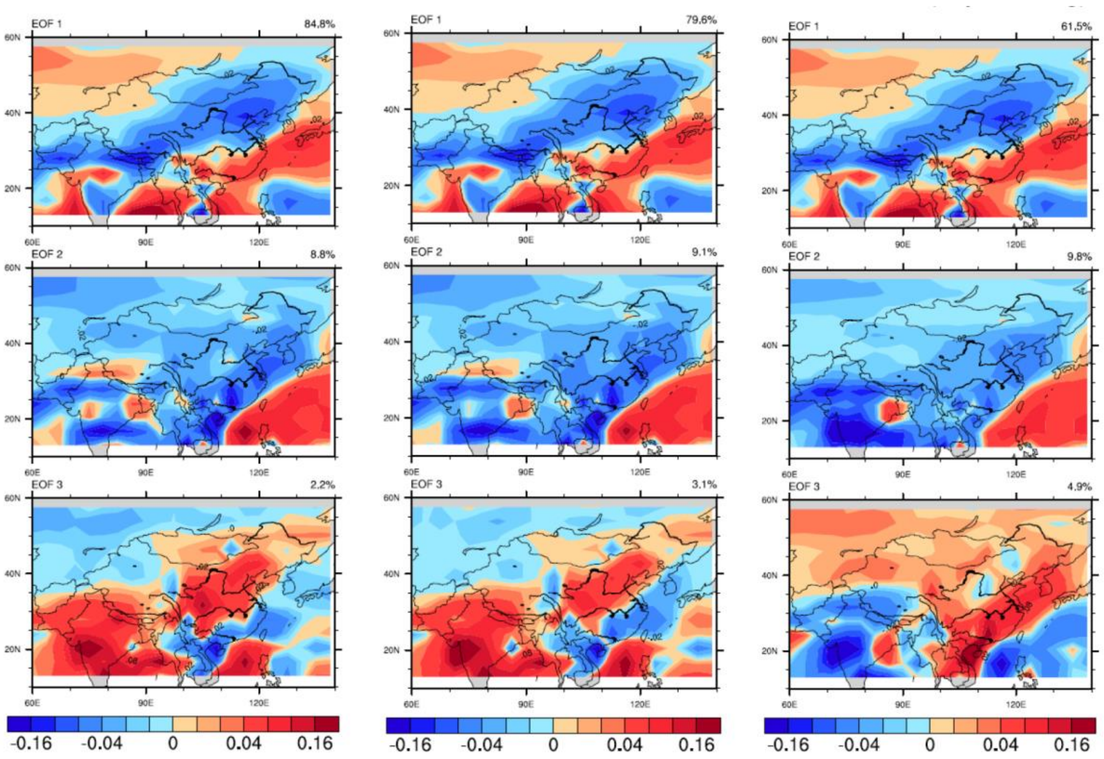

Fig. S16. The first 3 Empirical Orthogonal Function (EOF) modes of the summer (May–September) mean precipitation rate during the Holocene on the basis of 1000-yr running mean (left), 301-yr running mean (middle) and 31-yr running mean (right). The EOF analysis was performed on TRACE-21ka simulation results. It shows that summer precipitation in the Delingha region co-varies with that in Indian subcontinent and Northern China on various timescales from multidecadal to millennial, indicative of a common ASM circulation effect. This behaviour in these climate model simulations leads us to expect that Delingha precipitation in the real world would also co-vary with precipitation in other ASM-affected regions. General agreement in multi-millennial drying trends with proxy evidence of lower temporal resolution from stalagmite $\delta^{18}O$ records from eastern China, pollen-based precipitation reconstructions from eastern China and other moisture-sensitive proxy archives (Fig. 4b, 4c, SI Appendix, Figs. S12–S15) provides supportive evidence.

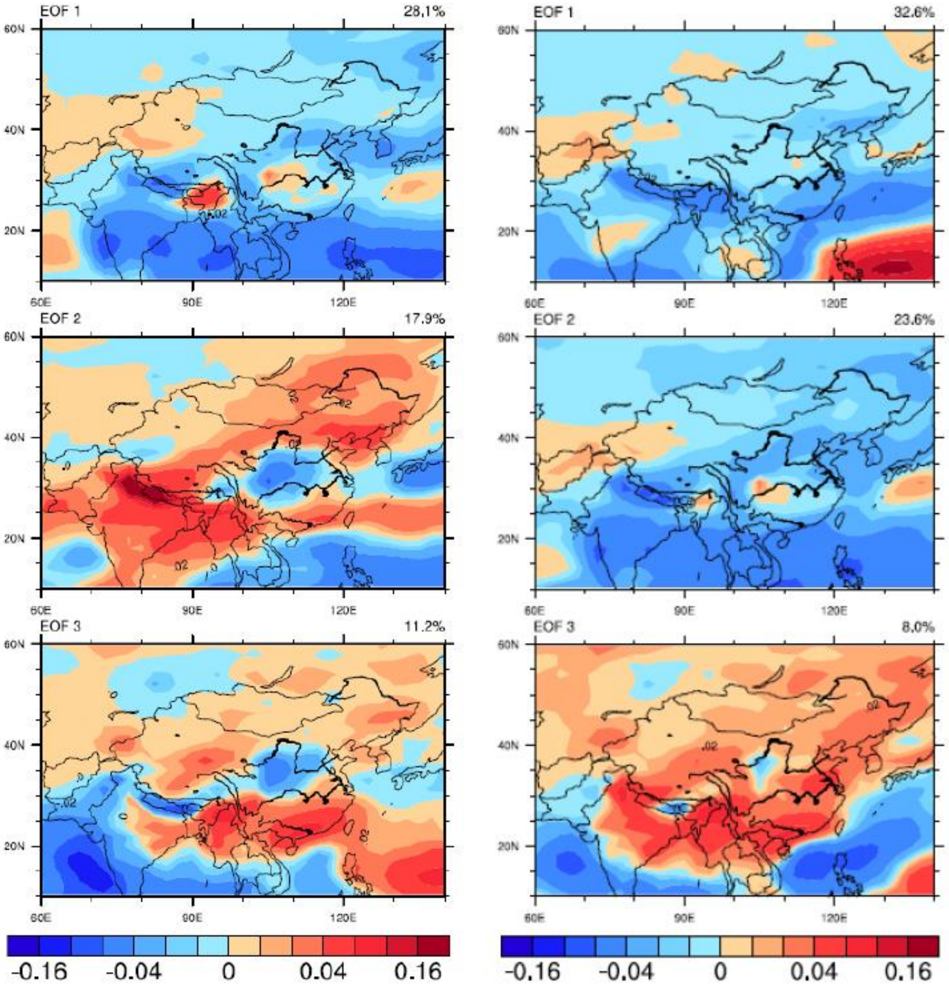

Fig. S17. The first 3 Empirical Orthogonal Function (EOF) modes of the summer (left, May-September) and annual (right) mean precipitation rate during 850–1850 CE on the basis of 31-yr running mean. The EOF analysis was performed on CESM-LME simulation results. It shows that summer/annual mean precipitation in the Delingha region co-varies with that in Indian subcontinent and Eastern China, indicative of a common ASM circulation effect.

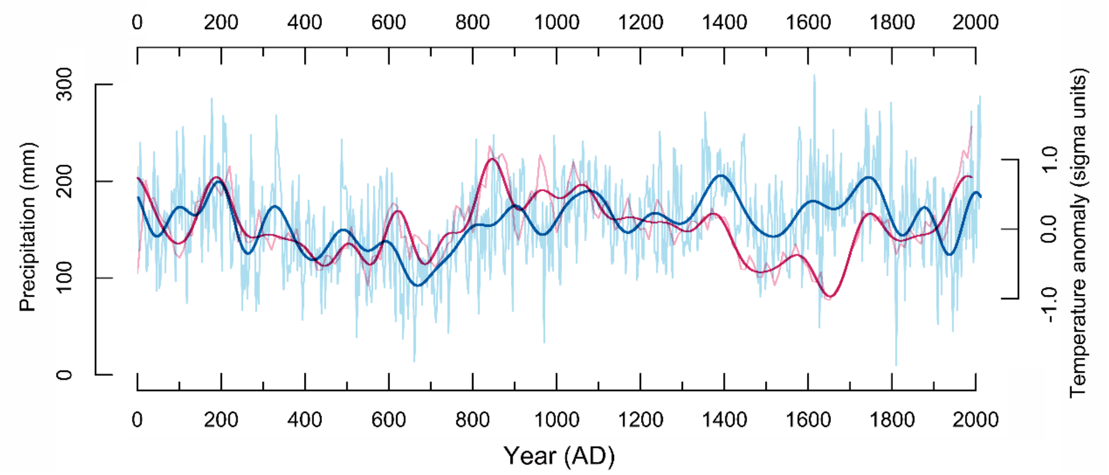

Fig. S18. Comparison of the DLH tree-ring $\delta^{18}O$ precipitation reconstruction (blue curve) with a temperature reconstruction for China (red curve) (61) spanning the past two millennia. The temperature reconstruction has a resolution of 10 years. Both series were first interpolated annually by using a linear interpolation method and then each series (thin line) was smoothed by a 100-point low-pass filter (heavy line) to highlight the century-scale variability. A statistical association after adjusting for degrees of freedom ($r=0.28$, $p<0.1$) with the centennial variability of reconstructed mean China-wide temperatures is detected over the past two millennia.

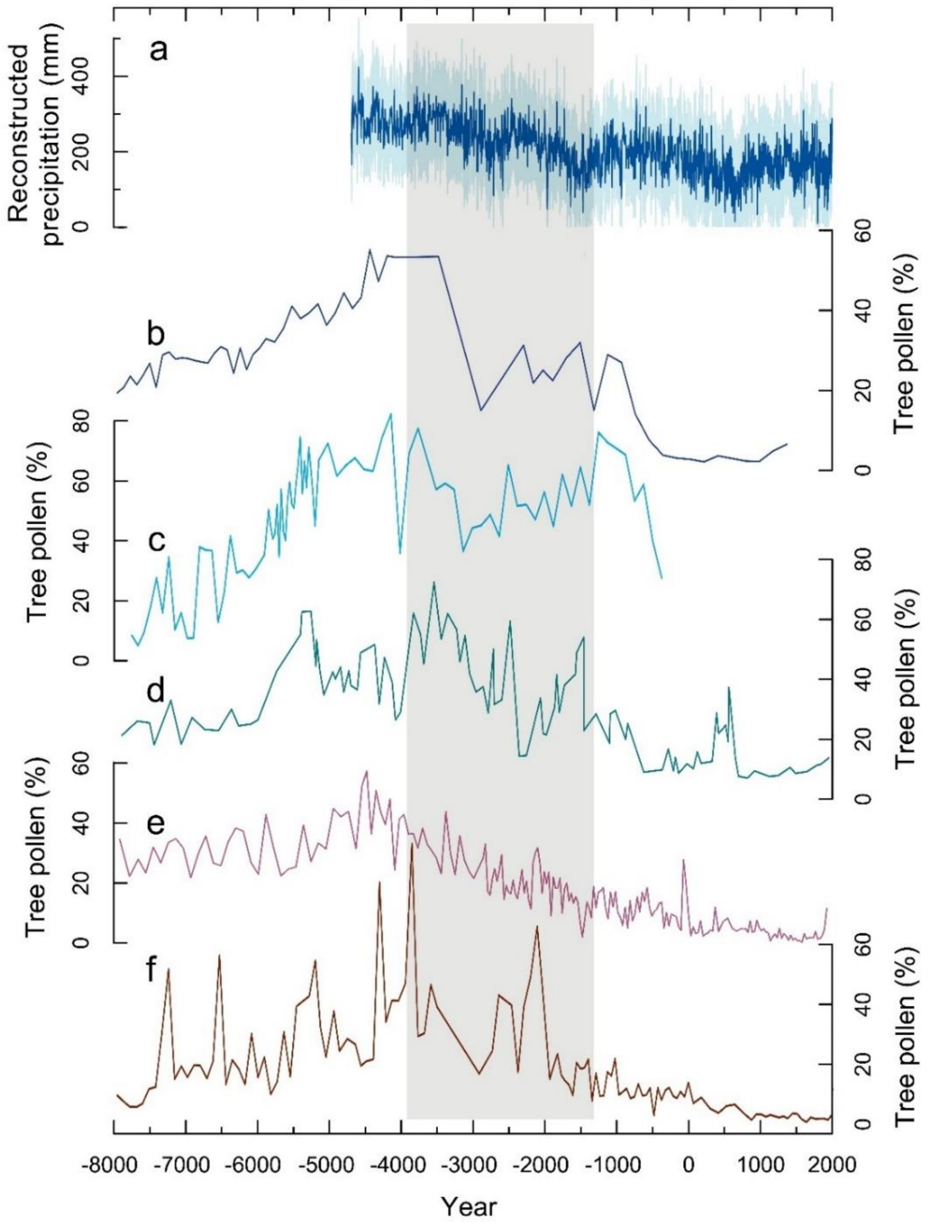

Fig. S19. (a) Tree-ring $\delta^{18}O$ precipitation reconstruction (DLH $\delta^{18}O$; this study); (b) tree pollen percentages from Bayanchagan Lake, northern China (52); (c) tree pollen percentages from Diaojiaohaizi Lake (53); (d) tree pollen percentages from Daihai Lake (54); (e) tree pollen percentages from Qinghai Lake (55); (f) tree pollen percentages from Dalianhai (56). The shaded area covers the common period from 4700–2000 BCE.

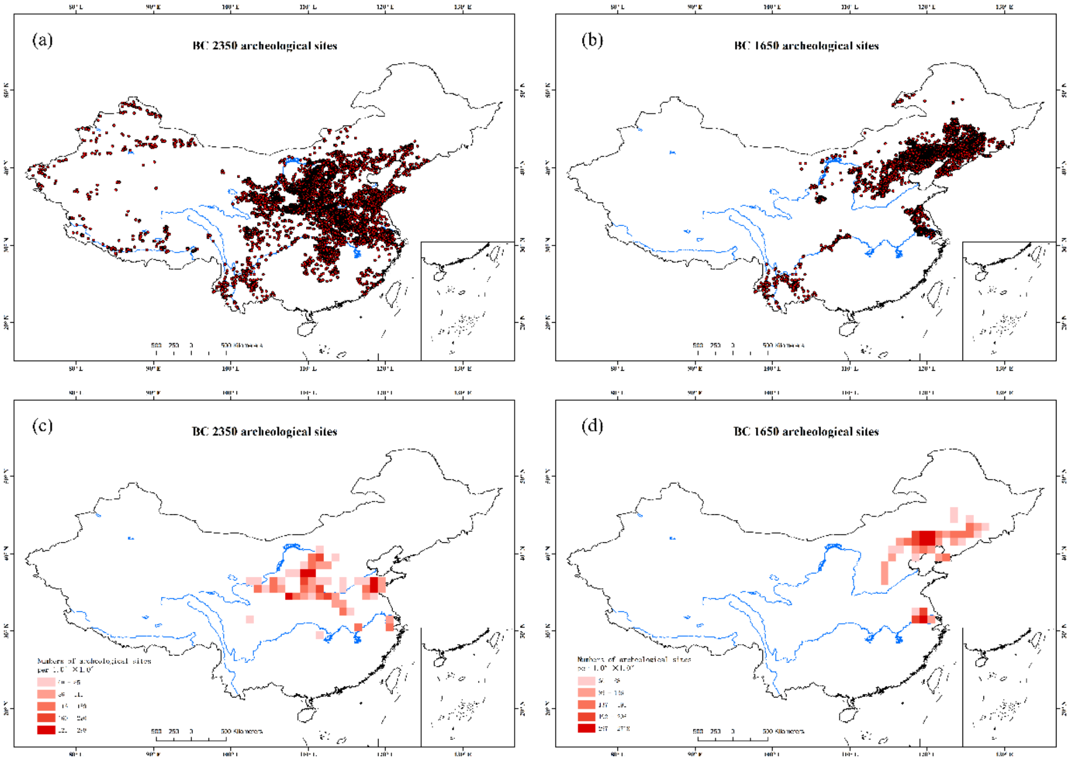

Fig. S20. Snapshots of archeological site dynamics in China in time under different climate conditions based on the digitalized archaeological dataset across China (57, 58). (a) The spatial distribution of archeological sites during a typical wet period (2350 BCE) in the Delingha isotope record. (b) The spatial distribution of archeological sites during a typical dry period (1650 BCE) in the Delingha isotope record. (c) Density of archeological sites in 2350 BCE. (d) Density of archeological sites in 1650 BCE. The site-density analysis includes all 1.0° × 1.0° grids with site number per grid exceeding 60. The areas with ⩽60 sites per grid remain blank. The maps show a pronounced shift in archaeological site densities from north-central China to northeastern China during 2350 BCE (a) and 1650 BCE (b).

REFERENCES

1. B. Yang et al., A 3,500-year tree-ring record of annual precipitation on the northeastern Tibetan Plateau. *Proc. Natl. Acad. Sci. U.S.A.* **111**, 2903–2908 (2014).
2. X. Shao et al., Climatic implications of a 3585-year tree-ring width chronology from the northeastern Qinghai-Tibetan Plateau. *Quat. Sci. Rev*. **29**, 2111–2122 (2010).
3. D. McCarroll, N. J. Loader, Stable isotopes in tree rings. *Quat. Sci. Rev*. **23**, 771–801 (2004).
4. M. Gagen, D. McCarroll, N. J. Loader, I. Robertson, *Dendroclimatology. Developments in Paleoenvironmental Research: Stable Isotopes in Dendroclimatology: Moving Beyond 'Potential'*, M. Hughes, et al., Eds. (Springer, 2011), pp. 147–172.
5. T. Wieloch et al., A novel device for batch-wise isolation of α-cellulose from small-amount wholewood samples. *Dendrochronologia* **29,** 115–117 (2011).
6. C. Qin et al., Drought signals in tree-ring stable oxygen isotope series of Qilian juniper from the arid northeastern Tibetan Plateau. *Glob. Planet. Chang*. **125**, 48–59 (2015).
7. C. Xu et al., Negligible local-factor influences on tree ring cellulose $\delta^{18}O$ of Qilian juniper in the Animaqing Mountains of the eastern Tibetan Plateau. *Tellus B Chem. Phys. Meteorol.* **69**, 1391663 (2017).
8. W. Wang et al., Moisture variations over the past millennium characterized by Qaidam Basin tree-ring $\delta^{18}O$. *Chin. Sci. Bull*. **58**, 3956–3961 (2013).
9. K. S. Treydte et al., The twentieth century was the wettest period in northern Pakistan over the past millennium. *Nature* **440**, 1179–1182 (2006).
10. J. E. Duffy et al., Absence of age-related trends in stable oxygen isotope ratios from oak tree rings. *Glob. Biogeochem. Cycles* **33**, 841–848 (2019).
11. J. Wernicke, J. Grießinger, P. Hochreuther, A. Bräuning, Variability of summer humidity during the past 800 years on the eastern Tibetan Plateau inferred from $\delta^{18}O$ of tree-ring cellulose. *Clim. Past* **11**, 327–337 (2015).
12. M. Sano et al., May–September precipitation in the Bhutan Himalaya since 1743 as reconstructed from tree ring cellulose $\delta^{18}O$. *J. Geophys. Res.* **118**, 8399–8410 (2013).
13. C. Xu et al., Potential utility of tree ring $\delta^{18}O$ series for reconstructing precipitation records from the lower reaches of the Yangtze River, southeast China. *J. Geophys. Res. Atmos*. **121**, 3954–3968 (2016).
14. T.M.L. Wigley, K.R. Briffa, P.D. Jones, On the average value of correlated time-series, with applications in dendroclimatology and hydrometeorology. *J. Climate Appl. Meteor.* **23**, 201–213 (1984).
15. X. Shao, L. Huang, H. Liu, Tree ring recorded millennial precipitation change in Delingha, Qinghai. *Sci. China Ser. D* **349**, 145–153 (2004).
16. K. R. Briffa et al., Tree-ring width and density data around the Northern Hemisphere: Part 1, local and regional climate signals. *Holocene* **12**, 737–757 (2002).
17. Z. Liu et al., Transient simulation of last deglaciation with a new mechanism for Bolling-Allerod warming. *Science* **325**, 310–314 (2009).
18. M. Yan, J. Liu, Physical processes of cooling and mega-drought during the 4.2 ka BP event: results from TraCE-21ka simulations. *Clim. Past* **15**, 265–277 (2019).

19. F. He, Simulating Transient Climate Evolution of the Last Deglaciation with CCSM3 (University of Wisconsin-Madison, 2011), 161p.
20. F. He et al., Northern Hemisphere forcing of Southern Hemisphere climate during the last deglaciation. Nature **494**, 81–85 (2013).
21. Z. Liu et al., Younger Dryas cooling and the Greenland climate response to CO_2. *Proc. Natl. Acad. Sci. U.S.A.* **109**, 11101–11104 (2012).
22. Z. Liu et al., Evolution and forcing mechanisms of El Nino over the past 21,000 years. *Nature* **515**, 550–553 (2014).
23. B. L. Otto-Bliesner, et al., Climate Variability and Change since 850 C.E.: An Ensemble Approach with the Community Earth System Model (CESM). *Bull. Amer. Meteor. Soc.* **5**, 735–754 (2016).
24. S. N. Rodionov, A sequential algorithm for testing climate regime shifts. *Geophys. Res. Lett.* **31**, L09204 (2004).
25. V. M. Muggeo, Segmented: an R package to fit regression models with broken-line relationships. *R News* **8**, 20–25 (2008).
26. R Core Team. *R: A Language and Environment for Statistical Computing. Vienna, Austria: R Foundation for Statistical Computing* (R Core Team, 2017).
27. R. Lu et al., A new find of macrofossils of Picea crassifolia Kom. in early–middle Holocene sediments of the Qinghai Lake basin and its paleoenvironmental significance. *Quat. Res*. **90**, 310–320 (2018).
28. L. G. Thompson et al., Abrupt tropical climate change: Past and present. *Proc. Natl. Acad. Sci. U.S.A.* **103**, 10536–10543 (2006).
29. M. Stebich et al., Holocene vegetation and climate dynamics of NE China based on the pollen record from Sihailongwan Maar Lake. *Quat. Sci. Rev*. **124**, 275–289 (2015).
30. Q. Xu et al., Pollen-based quantitative reconstruction of Holocene climate changes in the Daihai Lake area, Inner Mongolia, China. *J. Clim*. **23**, 2856–2868 (2010).
31. F. Chen et al., East Asian summer monsoon precipitation variability since the last deglaciation. *Sci. Rep*. **5**, 11186 (2015).
32. J. Li et al., Quantitative precipitation estimates for the northeastern Qinghai-Tibetan Plateau over the last 18,000 years. *J. Geophys. Res. Atmos*. **122**, 5132–5143 (2017).
33. J. Li et al., East Asian summer monsoon precipitation variations in China over the last 9500 years: a comparison of pollen-based reconstructions and model simulations. *Holocene* **26**, 592–602 (2016).
34. F. Lu et al., Variability of East Asian summer monsoon precipitation during the Holocene and possible forcing mechanisms. *Clim. Dyn*. **52**, 969–989 (2019).
35. Y. Goldsmith et al. Northward extent of East Asian monsoon covaries with intensity on orbital and millennial timescales. *Proc. Natl. Acad. Sci. U.S.A.* **114**, 1817–1821 (2017).
36. H. Long et al., Timing of Late Quaternary palaeolake evolution in Tengger Desert of northern China and its possible forcing mechanisms. *Glob. Planet. Chang*. **92**, 119–129 (2012).
37. F. Chen et al., Holocene vegetation history, precipitation changes and Indian Summer Monsoon evolution documented from sediments of Xingyun Lake, south-west China. *J. Quat. Sci*. **29**, 661–674 (2014).
38. Y. Wang, X. Liu, U. Herzschuh, Asynchronous evolution of the Indian and East Asian Summer Monsoon indicated by Holocene moisture pattern in monsoonal central Asia. *Earth Sci. Rev.* **103**, 135–153 (2010).
39. W. Wang, Z. Feng, Holocene moisture evolution across the Mongolian Plateau and its surrounding

areas: A synthesis of climatic records. *Earth Sci. Rev*. **122**, 38–57 (2013).
40. H. Wang, J. Chen, X. Zhang, F. Chen, Palaeosol development in the Chinese Loess Plateau as an indicator of the strength of the East Asian summer monsoon: Evidence for a mid-Holocene maximum. *Quat. Int*. **334**, 155–164 (2014).
41. H. Lu et al., Variation of East Asian monsoon precipitation during the past 21 ky and potential CO_2 forcing. *Geology* **41**, 1023–1026 (2013).
42. D. Xia et al., Out-of-phase evolution between summer and winter East Asian monsoons during the Holocene as recorded by Chinese loess deposits. *Quat. Res*. **81**, 500–507 (2014).
43. J. Laskar et al., A long-term numerical solution for the insolation quantities of the Earth. *Astron. Astrophys*. **428**, 261–285 (2004).
44. S. A. Marcott, J. D. Shakun, P. U. Clark, A. C. Mix, A reconstruction of regional and global temperature for the past 11,300 years. *Science* **339**, 1198–1201 (2013).
45. G. H. Haug et al., Southward migration of the intertropical convergence zone through the Holocene. *Science* **293**, 1304–1308 (2001).
46. J. Dong et al., Rapid retreat of the East Asian summer monsoon in the middle Holocene and a millennial weak monsoon interval at 9 ka in northern China. *J. Asian Earth Sci*. **151**, 31–39 (2018).
47. Y. Cai et al., The variation of summer monsoon precipitation in central China since the last deglaciation. *Earth Planet. Sci. Lett*. **291**, 21–31 (2010).
48. L. Tan et al., Centennial-to decadal-scale monsoon precipitation variations in the upper Hanjiang River region, China over the past 6650 years. *Earth Planet. Sci. Lett*. **482**, 580–590 (2018).
49. J. Dong et al., A high-resolution stalagmite record of the Holocene East Asian monsoon from Mt Shennongjia, central China. *Holocene* **20**, 257–264 (2010).
50. C. Hu et al., Quantification of Holocene Asian monsoon rainfall from spatially separated cave records. *Earth Planet. Sci. Lett*. **266**, 221–232 (2008).
51. Y. Wang et al., The Holocene Asian monsoon: links to solar changes and North Atlantic climate. *Science* **308**, 854–857 (2005).
52. W. Jiang et al., Reconstruction of climate and vegetation changes of Lake Bayanchagan (Inner Mongolia): Holocene variability of the East Asian monsoon. *Quat. Res*. **65**, 411–420 (2006).
53. C. Song, B. Wang, X. Sun, Implication of paleovegetational changes in DiaoJiao Lake Inner Mongolia. *Acta Bot. Sin*. **38**, 568–575 (1996).
54. J. Xiao et al., Holocene vegetation variation in the Daihai Lake region of north-central China: a direct indication of the Asian monsoon climatic history. *Quat. Sci. Rev*. **23**, 1669–1679 (2004).
55. J. Shen, X. Liu, S. Wang, R. Matsumoto, Palaeoclimatic changes in the Qinghai Lake area during the last 18,000 years. *Quat. Int.* **136**, 131–140 (2005).
56. B. Cheng, F. Chen, W. Zhang, Palaeovegetational and palaeoenvironmental changes since the last deglacial in Gonghe Basin, northeast Tibetan Plateau. *J. Geogr. Sci.* **23**, 136–146 (2013).
57. M. Wagner et al., Mapping of the spatial and temporal distribution of archaeological sites of northern China during the Neolithic and Bronze Age. *Quat. Int*. **290**, 344–357 (2013).
58. D. Hosner et al., Spatiotemporal distribution patterns of archaeological sites in China during the Neolithic and Bronze Age: An overview. *Holocene* **26**, 1576–1593 (2016).
59. X Liu et al., Spatial-temporal variability and climatic significance of tree ring's carbon isotopes of *picea crassifolia* on the Qilian Mountains. *J. Glaciol. Geocryol.* **4, 666-676** (2010).
60. Q. Sun et al., Climate as a factor for Neolithic cultural collapses approximately 4000 years BP in China. *Earth Sci. Rev.* **197**, 102915 (2019).

61. B. Yang, A. Braeuning, K.R. Johnson, Y.F. Shi, General characteristics of temperature variation in China during the last two millennia. *Geophys. Res. Lett.* **29**, 1324 (2002).
